# Optimized SPE‐HPLC‐FLD Method for the Simultaneous Determination of Olaparib, Propranolol, and Furosemide in Human Urine

**DOI:** 10.1002/bmc.70529

**Published:** 2026-06-26

**Authors:** Georgios Kamaris, Antonia Kalagia, Catherine K. Markopoulou

**Affiliations:** ^1^ Laboratory of Pharmaceutical Analysis, Department of Pharmacy Aristotle University of Thessaloniki Thessaloniki Greece

**Keywords:** furosemide, olaparib, propranolol, solid‐phase extraction (SPE), urine

## Abstract

Olaparib belongs to the PARP inhibitors and is widely used as an anticancer drug. Due to frequent side effects, it is usually co‐administered with an antihypertensive, such as propranolol and/or an antidiuretic, such as furosemide. In this study, a highly sensitive HPLC‐FLD method was developed for the simultaneous determination of the three APIs in urine. For their analysis, a Nucleosil C8 column (250 mm × 4.6 mm, 5 μm) at 50°C was used as the stationary phase and an isocratic system of methanol–acetonitrile–20 mM NaH_2_PO_4_ at pH 3.6, 12:20:68 v/v/v as the mobile phase. The calibration curves for the analytes were generated both in urine and in the pure diluent. The reliability of the method was evaluated according to ICH guidelines in terms of accuracy and repeatability. For sample purification, protein precipitation techniques involving the addition of organic solvents, acids, or salts, as well as the freezing‐out technique, were studied. Liquid–liquid extraction, using various solvents, and solid‐phase extraction (SPE) were also tested, where the latter was found more suitable. SPE optimization was achieved using experimental design and a D‐optimal mixture design. Reliable analyte recoveries demonstrate the capability of the proposed method, making it a suitable tool for biological studies.

## 1 Introduction

Despite the undeniable progress of science, cancer is a disease that affects a large portion of the population. A particular type of cancer is associated with BRCA1 and BRCA2 mutations, which have been shown to increase the risk of cancers of the breast, ovaries, pancreas, and prostate (Li et al. 2022). The incidence of BRCA1 and BRCA2 genes in breast cancer is 5%–7% and can reach up to 90% in hereditary cases (Gui 2025).

Given the severity of the problem, there are many drug categories that have been developed to combat it. Poly‐(ADP‐ribose) polymerase (PARP) inhibitors are a new class that exploit defects in the DNA repair mechanisms of cancer cells with BRCA mutations, ultimately leading them to cell death. An important representative of the PARP inhibitors is olaparib (OLA) (Knox et al. 2024). However, PARP‐1–based therapy is associated with a significantly higher risk of major adverse cardiac events, hypertension, and thromboembolic symptoms (Palazzo et al. 2023). Thus, although OLA (150 mg/tablet) causes fewer problems than other PARP inhibitors, its adverse effects, such as hypertension (especially when administered in doses greater than 800 mg/day), are important and must be addressed (Chen et al. 2023). According to the World Health Organization (WHO), approximately 1.28 billion adults aged 30–79 years worldwide suffer from hypertension (“Hypertension,” 2025). Some of them are also cancer patients and need simultaneous anticancer and antihypertensive treatment. Two widely used classes of active pharmaceutical ingredients (APIs) against blood pressure are beta‐blockers and diuretics. Beta‐blockers are sympatholytic drugs that act as antagonists in the binding of adrenaline and noradrenaline to beta‐adrenergic receptors, resulting in a decrease in heart rate and blood pressure (Chachlioutaki et al. 2025). Diuretics increase urine excretion by reducing the amount of fluid and electrolytes (such as sodium) in the body, which also leads to a decrease in blood pressure.

Of particular interest are some epidemiological studies and clinical trials that indicate the positive effects of beta‐blockers in inhibiting cancer progression (Cavalu et al. 2024). Among them, propranolol (PRP), which is administered in the form of intravenous solutions (1 mg mL^−1^), oral capsules, and extended‐release tablets (dose: 120, 160, 60, 80 mg), has the most important role in BRAC cancers (Pantziarka et al. 2016). Furosemide (FUR) belongs to the loop diuretics and acts by inhibiting the reabsorption of Na^+^‐K^+^‐2Cl^−^ (Di Fulvio et al. 2025). Similarly, this drug seems to contribute to the reversal of the multidrug resistance (MDR) state in bladder cancer cell lines (Speers et al. 2006). Therefore, both PRP and FUR are administered for the treatment of hypertension, but due to their synergistic action with OLA, they are also proposed as a therapeutic regimen in cancer patients.

The APIs PRP, OLA, and FUR exert their therapeutic effects primarily in their unmetabolized form (Gulsun et al. 2025; Luketic and Sanyal 2000; Miura et al. 2012). As metabolism occurs predominantly in the liver, the induction or inhibition of specific enzymes can alter the rate of metabolism and thereby indirectly influence the metabolism of other co‐administered agents (i.e., through drug–drug interactions). Cancer cells can also modify the metabolism or distribution of drugs, thereby reducing effective drug concentrations (Zhu et al. 2025). More specifically, PRP is metabolized mainly via CYP2D6, CYP1A2, and CYP2C19 and OLA via CYP3A4/5, whereas FUR undergoes glucuronidation and is hepatic to a lesser extent (Hedera et al. 2013; Lapini et al. 2023; Sandré et al. 2024).

According to recent pharmacokinetic studies, under normal physiological conditions, all three APIs can be detected in urine both as metabolites and as unchanged compounds. When administered as monotherapy, OLA is excreted in urine at approximately 44%, with 15% recovered as the free (unmetabolized) form (Bruin et al. 2022). FUR exhibits particularly high renal elimination, reaching up to 65% of the administered dose (Huang et al. 2016). In contrast, free PRP, whose main active metabolite is hydroxy‐PRP, shows the lowest urinary excretion rate, only 1%–4% (Shukla et al. 2009). However, co‐administration of these substances may significantly alter their metabolic rates, either through competition for the same enzymatic pathways or through additional burden on hepatic function.

Moreover, substantial variability in drug excretion is observed under real clinical conditions, as actual patient populations are far more heterogeneous than those enrolled in controlled clinical trials (e.g., age, sex, body weight, comorbidities, and concomitant medications) (Ferrer et al. 2024). In particular, cancer patients represent a highly vulnerable group, often presenting with multiple comorbidities due to polypharmacy (Lanser et al. 2023). Although therapeutic drug monitoring (TDM) is an established practice in other pharmacological classes (e.g., antibiotics), its application in oncology remains limited. In the context of polypharmacy, assessing the individualized pharmacokinetic profile of each patient and tailoring a personalized therapeutic scheme becomes essential, considering metabolic interactions and variations in hepatic function. Within this framework, urine analysis of PRP, OLA, and FUR can play a pivotal role in the development of specialized therapeutic strategies, contributing to treatment individualization, optimization of therapeutic efficacy, and reduction of adverse effects.

The analysis of biological samples is a key tool in biomedical research and diagnostic medicine in the field of quality control. Urine is one of the most common substrates, which, due to its significant advantages, has been studied with various analytical methods. Urine samples are easily accessible and are available in large quantities, as their collection is noninvasive and does not require specialized personnel. It is worth noting that the average urine production of a healthy person ranges from 1 to 2 L/day. Furthermore, urine samples provide important information about the body's metabolic processes, the identification of biomarkers of various diseases, and the evaluation of the pharmacokinetics of drugs (Alampanos and Samanidou 2021; Catanese et al. 2024; Meister et al. 2021; Patakidou et al. 2025).

International literature reports a significant number of analytical methods for the chromatographic determination of the three active substances in complex substrates. The most relative are briefly presented in Table 1. None of them simultaneously determines OLA, PRP, and FUR, despite their frequent co‐administration in complex therapeutic schemes, while the combination of the two antihypertensives has been studied mainly with HPLC and MS or UV detectors (Alothman et al. 2020; Derwand et al. 2024; Soltani and Jouyban 2010; Zhao et al. 2012).

**TABLE 1:** Literature review for determination of OLA, PRP, and FUR.

Reference title	Comments
“QuEChERS as Alternative Extraction Procedure in Doping Analyses” (Derwand et al. 2024)	HPLC–MS/MS, matrix: Urine, St.Ph: ZORBAX Eclipse XDB‐C8 column (3.5 μm, 2.1 mm, 100 mm, Agilent Technologies) M.Ph: Gradient, A: 2 mM CH_3_COONH_4_, 0.1% CH_3_COOH (v/v)/ACN (95:5, v/v); B: CH_3_COONH_4_, 0.1% CH_3_COOH (v/v)/ACN (5:95, v/v) LOI (limit of identification): FUR 5 ng mL^−1^, PRP 12.5 ng mL^−1^ Pr: QuEchERS
“Applications of Shun Shell Column and Nanocomposite Sorbent for Analysis of Eleven Anti‐Hypertensive in Human Plasma” (Alothman et al. 2020)	HPLC‐UV, matrix: Plasma, St.Ph: Shun Shell C18 150 × 4.6 mm, 2.6 μm M.Ph: CH_3_COONH_4_ pH 5.5–MeOH–ACN (65:18:17) LOD: FUR 1.5 μg L^−1^, PRP 0.10 μg L^−1^ LOQ: FUR 14.67 μg L^−1^, PRP 1.0 μg L^−1^ Pr: Solid‐phase micro membrane tip extraction (SPMMTE)
“Chemometric Resolution of Coeluting Peaks of Eleven Antihypertensives From Multiple Classes in High Performance Liquid Chromatography: Comprehensive Research in Human Serum, Health Product and Chinese Patent Medicine Samples” (Zhao et al. 2012)	HPLC‐DAD, matrix: Human serum St.Ph: WondaSil C18 column (5 m, 150 mm × 4.6 mm, GL Sciences Inc., Japan) M.Ph: MeOH and 10 mM KH_2_PO_4_ (pH 2.60),58:42 (v/v) LOD: FUR 0.17 μg mL^−1^, PRP 0.052 μg mL^−1^ LOQ: FUR 0.50 μg mL^−1^, PRP 0.16 μg mL^−1^ Pr: Protein precipitation with methanol
“Optimization and Validation of an Isocratic HPLC‐UV Method for the Simultaneous Determination of Five Drugs Used in Combined Cardiovascular Therapy in Human Plasma” (Soltani and Jouyban 2010)	HPLC‐UV, matrix: Plasma St.Ph: C18 ODS‐3 MZ‐l (15 mm × 4.6 mm, 5 μm) M.Ph: ACN/2‐propanol/15 mM phosphate buffer (pH 2) (32.5/2.5/65 v/v) ULOQ (upper limit of quantification): FUR 0.8 μg mL^−1^, PRP 0.8 μg mL^−1^ LLOQ (lower limit of quantification): FUR 0.025 μg mL^−1^ PRP 0.025 μg mL^−1^ Pr: Protein precipitation with acetonitrile and zinc sulfate
“Simultaneous High‐Performance Liquid Chromatographic Assay of Furosemide and Propranolol HCL and Its Application in a Pharmacokinetic Study” (El‐Saharty 2003)	HPLC‐UV, matrix: Plasma St.Ph: Nucleosil C18 (250, 4.6 mm i.d., particle size 10 mm) M.Ph: 0.02 M KH_2_PO_4_–ACN, 80:20 v/v pH 4.5 LOD: FUR 0.04 μg mL^−1^, PRP 1 μg mL^−1^ LOQ: FUR 0.1 μg mL^−1^, PRP 5 μg mL^−1^ Pr: Protein precipitation with acetonitrile and liquid–liquid extraction (LLE) with diethyl acetate
“Development and Comparative Evaluation of Two Different Label‐Free and Sensitive Fluorescence Platforms for Analysis of Olaparib: A Recently FDA‐Approved Drug for the Treatment of Ovarian and Breast Cancer” (Darwish and Khalil 2023)	HPLC‐FD, matrix: Plasma St.Ph: Nucleosil‐CN (250 mm length × 4.6 mm i.d., 5 μm) M.Ph: Acetonitrile:water (25:75, v/v) LOD: OLA 1.7 ng mL^−1^, LOQ: OLA 5 ng mL^−1^ Pr: Protein precipitation with methanol
“Quantitative Characterization of Olaparib in Nanodelivery System and Target Cell Compartments by LC–MS/MS” (Ottria et al. 2019)	HPLC–MS/MS, matrix: Urine St.Ph: C18 column (2.1 × 150 mm, 3 μm, precolumn C18 cartridge (4 × 3.0 mm) M.Ph: 10 mM HCOONH_4_, 0.1% HCOOH (A) and ACN (B) LOD: OLA 0.02 ng mL^−1^, LOQ: OLA 0.12 ng mL^−1^ Pr: Protein precipitation with acetonitrile
“Validated Stability Indicating Assay Method of Olaparib: LC‐ESI‐Q‐TOF‐MS/MS and NMR Studies for Characterization of Its New Hydrolytic and Oxidative Forced Degradation Products” (Thummar et al. 2018)	HPLC–MS/MS St.Ph: Inert sustain C18 (250 × 4.6 mm, 5 μm) M.Ph: Gradient elution: 10 mM CH_3_COONH_4_ (pH 4.5)/ACN LOD: OLA 0.79 μg mL^−1^, LOQ: OLA 2.48 μg mL^−1^
“Development and Validation of a High‐Performance Liquid Chromatography Method for the Quantitation of Intracellular PARP Inhibitor Olaparib in Cancer Cells” (Daumar et al. 2018)	HPLC‐DAD St.Ph: Nova‐Pak 4‐μm C18 column (150 × 3.9 mm; Waters and Nova‐Pak guard column) M.Ph: Gradient elution: ACN/water LOD: OLA 50 ng mL^−1^, LOQ: OLA 200 ng mL^−1^ Pr: Protein precipitation with acetonitrile and cell lysis
“Micro‐Extraction by Packed Sorbent Coupled On‐Line to a Column‐Switching Chromatography System—A Case Study on the Determination of Three Beta‐Blockers in Human Urine” (Šatínský et al. 2019)	HPLC‐FLD, matrix: Urine St.Ph: Triart YMC C‐18, 50 mm × 4.6 mm, 5 μm M.Ph: Gradient elution: ACN/0.5% triethylamine with CH_3_COOH, pH 4.5 LOD: PRP 1.4 ng mL^−1^, LOQ: PRP 4.6 ng mL^−1^ Pr: Micro‐extraction by packed sorbent
“Determination of Propranolol Concentration in Small Volume of Rat Plasma by HPLC‐FLD” (Kim et al. 2001)	HPLC‐FLD, matrix: Rat plasma St.Ph: 5 mm CAPCELL PAK CN UG1201 (250, 4.6 mm i.d.) M.Ph: 1%—CH_3_COOH (1%) with 0.2% triethylamine and ACN (65:35 v/v; pH 3.8) LOD: PRP 1.34 ng mL^−1^, LOQ: PRP 2 ng mL^−1^ Pr: Protein precipitation with acetonitrile
“A Fluorimetric Liquid Chromatographic Method for the Determination of Propranolol in Human Serum/Plasma” (Rekhi et al. 1995)	HPLC‐FLD, matrix: Plasma/serum St.Ph: Hypersil ~ CN column (250 × 4.6 mm Z; 5 t.tm) M.Ph: ACN–CH_3_COOH (1%) with 0.2% triethylamine (35:65, v/v) (pH 3.6) LOD: PRP 2 ng mL^−1^, LOQ: PRP 5 ng mL^−1^ Pr: Protein precipitation with acetonitrile
“Monitoring of Biogenic Amines and Drugs of Various Therapeutic Groups in Urine Samples With Use of HPLC” (Baranowska and Płonka 2016)	HPLC‐FLD, matrix: Urine St.Ph: LiChroCARD Purospher RP18, 125 × 3 mm, 5 μm M.Ph: Gradient elution: Acetate buffer (pH 4.66)/MeOH LOD: FUR 50 ng mL^−1^, LOQ: FUR 150 ng mL^−1^ Pr: Solid‐phase extraction (SPE)
“High‐Throughput Liquid‐Chromatography Method With Fluorescence Detection for Reciprocal Determination of Furosemide or Norfloxacin in Human Plasma” (Galaon et al. 2007)	HPLC‐FLD, matrix: Plasma St.Ph: monolithic RP‐18 (100 mm, 4.6 mm) M.Ph: 0.015 mol L^−1^ sodium heptanesulfonate, 0.2% triethylamine and H_3_PO_4_ pH 2.5‐CAN‐MeOH 70:15:15 (v/v/v) LOD: FUR 8.2 ng mL^−1^, LOQ: FUR 27 ng mL^−1^ Pr: Protein precipitation with acetonitrile
“Determination of Furosemide in Plasma and Urine Using Monolithic Silica Rod Liquid Chromatography” (Wenk et al. 2006)	HPLC‐FLD, matrix: Plasma, urine St.Ph: RP 18 (100 mm × 4.6 mm) Chromolith monolithic M.Ph: Gradient, A: (20% acetonitrile), B: (80% ACN), both in 0.025% CH_3_COOH LLOD: FUR 3 ng mL^−1^, LLOQ: FUR 12 ng mL^−1^ Pr: Protein precipitation with hydrochloric acid and LLE with diethyl ether
“Simultaneous Determination of 11 Drugs Belonging to Four Different Groups in Human Urine Samples by RP‐HPLC Method” (Baranowska et al. 2006)	HPLC‐FLD, matrix: Urine St.Ph: LiChroCART C18 (125 mm × 3 mm, particle size 5 m) M.Ph: 0.05% TFA (A), MeOH (B), and ACN (C), gradient elution LOD: FUR 0.02 μg mL^−1^, LOQ: FUR 0.05 μg mL^−1^ Pr: Protein precipitation with acetonitrile and methanol
“Method Validation for Determination of Furosemide in Plasma Liquid–Liquid Extraction and HPLC‐FLD” (Gomez et al. 2005)	HPLC‐FLD, matrix: Plasma St.Ph: Kromasil 100‐5C18 (5 mm, 150 × 4.6 mm) M.Ph: 0.02 M KH_2_PO_4_–ACN (34:66 v/v), pH to 3.0 LOD: FUR 0.001 μg mL^−1^, LOQ: FUR 0.003 μg mL^−1^ Pr: Protein precipitation with acetonitrile and LLE with ethyl acetate

Abbreviations: St. Phase: stationary phase, M. Ph.: mobile phase, Pr: pretreatment.

A common feature of most studies is that special attention is paid to the purification of the substrate, whether it is a biological sample or drug excipients. This process plays a critical role in any bioanalytical method, as it allows the selective isolation of the target analyte from the matrix, reduces or removes interfering components, and allows the preconcentration of the API. To achieve sample cleanup, various techniques have been used, such as protein precipitation, liquid–liquid extraction (LLE), and solid‐phase extraction (SPE) or even a combination of these. Protein precipitation, which can be succeeded with an organic solvent, acid, salt, or even a metal ion, is commonly used to quickly clean up samples and disrupt the protein–drug binding. It is a simple process that can also be a precursor stage for further processing (Polson et al. 2003). LLE, although not selective, does not require specialized equipment (significantly reducing costs) and is considered an easy‐to‐perform purification method (Rawa‐Adkonis et al. 2006). Finally, SPE ensures high recoveries and excellent repeatability while offering the possibility of pre‐concentration of the analyte, improving sensitivity. In addition, the extract obtained is cleaner, with minimized interferences and components of the biological substrate. The method is also particularly attractive in bioanalytical applications, as it requires smaller amounts of organic solvents and facilitates the automated processing of many samples simultaneously (Badawy et al. 2022).

In the present study, a new reliable and highly sensitive HPLC–FLD method was developed and validated, suitable for the simultaneous determination of OLA, FUR, and PRP (trace analysis) in urine. Emphasis was placed on the sample cleanup process and the quantitative recovery of APIs from the substrate. Indicatively, the protein precipitation by the addition of organic solvents or acids or salt (salting out) was tested. For the recovery of the analytes, LLE, SPE, and their combination were also examined. SPE was considered as the optimal method and was further studied using cartridges of different types and technical characteristics. Finally, it was optimized using experimental design and the D‐optimal mixture methodology. The SPE technique was reproducible and easy to use over a wide range of concentrations for OLA, FUR, and PRP. In conclusion, the proposed HPLC‐FLD analytical procedure can reliably describe the urinary excretion rate of the three active ingredients and serve as a tool for administering the optimal therapeutic regimen.

## 2 Materials and Methods

### 2.1 Reagents and Solvents

Acetonitrile (ACN) and methanol, both of HPLC gradient grade, were purchased from VWR Chemicals (Radnor, PA, USA). High‐purity water (18.2 MΩ cm resistivity) was obtained using a B30 water purification system (Adrona SIA, Riga, Latvia). Formic acid (FA; Sigma‐Aldrich, St. Louis, MI, USA) was added to the mobile phase at a concentration of 0.1%. Phosphate buffer (20 mM, pH 3.6) was prepared by adding 2.8 g of NaH_2_PO_4_ (Merck, Darmstadt, Germany) to 1 L of water. The pH of the phosphate buffer was adjusted using phosphate acid.

PRP (TCI, Zwijndrecht, Belgium, purity 99%), FUR (Fagron, Rotterdam, Netherlands, purity 99.1%), and OLA (BLD pharm, Kaiserslautern, Germany, purity 98%) were used as reference standards.

### 2.2 Stock Solutions

An accurately weighed amount of 5.0 mg of FUR, PRP, and OLA was dissolved separately in 10‐mL volumetric flasks filled with MeOH. A mixed standard solution containing the three analytes was then prepared (100 ng mL^−1^ FUR, 100 ng mL^−1^ PRP, and 200 ng mL^−1^ OLA), and through appropriate serial dilutions, two sets of six calibration standards (1–100 ng mL^−1^ FUR, 1–100 ng mL^−1^ PRP, and 1–200 ng mL^−1^ OLA) were obtained in the diluent and urine substrates.

### 2.3 Sample Pretreatment

For the processing of urine samples, the SPE technique was applied using C18 Supelclean‐SPE cartridges of 3 mL. According to the procedure, conditioning was carried out with 2 mL MeOH, 1 mL H_2_O and 1 mL 0.5% FA. The cartridge was then loaded with 1.0 mL of urine spiked with different concentrations of analytes at pH 3.6 (with the addition of 50 μL of NaH_2_PO_4_ at 350 mM) and washed with 1 mL 0.5% FA. The APIs were eluted with a 1.0‐mL mixture, containing an isopropanol (ISO)–ACN–phosphate buffer (50 mM, pH 5.4), 64:27:9 v/v. The obtained eluate was analyzed with the proposed HPLC–FLD method.

### 2.4 Instruments and Equipment

Chromatographic analyses were conducted on a Shimadzu HPLC system equipped with two LC‐20ad pumps, a DGU‐14A degasser, a SIL‐10ad autosampler (injection volume: 30 μL), and an RF‐20A fluorescence detector (Shimadzu, Tokyo, Japan). The detector was operated at a gain setting of ×4, with high sensitivity. Detection of the analytes was achieved using specific excitation/emission wavelength pairs (λexc/λem) of 240/350 nm for OLA, 232/675 nm for PRP, and 327/412 nm for FUR.

Separation was achieved on a reversed‐phase Nucleosil Macherey‐Nagel C8 column (250 mm × 4.6 mm, 5 μm), maintained at 50°C in a CTO‐10AS VP column oven (Shimadzu, Tokyo, Japan). To carry out the analysis, isocratic elution (a flow rate of 1.0 mL min^−1^) was used with a mobile phase consisting of three solvents: (A) methanol, (B) ACN, and (C) NaH_2_PO_4_ 20 mM buffer, pH 3.6, at a ratio of 12:20:68 v/v/v.

## 3 Results and Discussion

### 3.1 Development of the Chromatographic Method

The aim of this investigation was to propose a reliable and sensitive analytical method that would ensure the simultaneous determination of the three analytes in urine. The problems that had to be addressed were elution of the first peak (FUR) away from the solvent front (urine substrate), while keeping the overall run time relatively short. At the same time, due to the high sensitivity of the FLD detector, it was desirable that the mobile‐phase elution system be isocratic, in order to ensure baseline stability. Equally important was the improvement of the chromatographic peak shape, especially of FUR, which in some cases was double. Thus, both the ratio of solvents and pH of the mobile phase were examined on a case‐by‐case basis, using a series of reversed‐phase columns (‐phenyl, ‐C18, ‐CN, and ‐C8), as described below.

Starting with a Discovery HS C18 column (150 mm × 4.6 mm, 5 μm) and a mobile phase of MeOH–FA 0.1% at 40:60 v/v, the coelution of FUR and PRP was observed at 10 min, while OLA was eluted at 13 min. Since changes in the mobile phase ratio did not improve the FUR–PRP separation, the effect of pH was also examined using a 20 mM NaH_2_PO_4_ buffer. At pH 6.8, FUR was eluted with the solvent front, due to its ionization, while the problem was not corrected at a lower pH of up to 4.5. Finally, at a pH of 2.7, FUR was removed from the solvent front but partially overlapped with the PRP. Therefore, the Discovery Supelco HS C18 column (150 mm × 4.6 mm, 5 μm) was discarded, and other columns of longer length (250 mm) and a different retention mechanism were tested. When applying the Phenyl‐ACE (250 mm × 4.6 mm, 5 μm) column at pH 6.8 and with a mobile phase of MeOH:buffer NaH_2_PO_4_, 40:60, FUR was eluted after the solvent front and was completely separated from the other two analytes at a total run time of 15 min. However, in this case, the problem was the peak shape for both FUR and PRP (tailing factor [Tf] > 2), which was not corrected even by replacing methanol with ACN. Alternatively, a low pH value (at 2.7) of the mobile phase was tested with either 0.1% FA or a buffer solution. In this case, FUR was further removed from the solvent front (non‐ionized form) giving good separations with the remaining analytes, but the problem of the double peak and high Tf values remained, making the column unsuitable. Similar behavior was observed with the CN Waters Spherisorb (250 mm × 4.6 mm, 5 μm) column, which was rejected due to the peak shape of FUR and PRR. At pH 3.6 (with 20 mM NaH_2_PO_4_ buffer), the FUR had a double peak, while PRP had Tf > 2. When a mobile phase with 0.1% FA was used, the problem was not improved.

Finally, the Nucleosil Macherey‐Nagel C8 column (250 mm × 4.6 mm, 5 μm) was selected as the optimal one, since it presented the best chromatographic separations. However, its use had to be further investigated regarding the composition and pH of the mobile phase.

A general observation, regarding the composition of the mobile phase, was the necessity of buffer salts on the aqueous solution, so that the chromatographic peaks of the analytes be isometric and not double. The investigation of pH was carried out taking into account the molecular structures (Figure S1) and the physicochemical properties of the actives. As shown in Table S1, although the three substances have both acidic and basic groups in their structure, due to their pKa values they are stably ionized. Only FUR can appear in its ionized and non‐ionized form, depending on the pH of the mobile phase (pKa_strongest acid_ = 4.25) (Wishart et al. 2022).

Regarding the organic solvents of the mobile phase, methanol (MeOH) and ACN were examined, individually and in mixtures in various proportions. It is worth noting that, unlike ACN, the elution power of methanol was not equivalent for both substances (FUR and PRP). That is, the reduction of MeOH did not cause a corresponding delay in the elution of FUR and PRP, resulting in overlapping of their peaks. After further investigation, a mobile phase consisting of MeOH‐ NaH_2_PO_4_ 20 mM at pH = 3.6, 45:55 v/v was considered sufficient. In this case, however, there was a very high backpressure in the system, which could, in part, be corrected by increasing the column temperature (50°C) and decreasing the flow rate (0.8 mL min^−1^) (Figure S2). Nonetheless, the given analytical conditions continued to burden the system over time. Alternatively, a mobile phase with gradient elution was used, in which the methanol concentration started at 20% and reached 45% within the first five minutes (T = 45°C and flow rate 1 mL min^−1^). Although the proposed chromatographic separation resulted in a lower system pressure, the use of an HPLC‐FLD setup is not recommended when the detector is used at high sensitivity (unstable baseline). On the contrary, it is considered ideal for a UV detector and routine analyses (Figure S3).

Thus, to solve the problem, the case of replacing MeOH, in the mobile phase, with ACN was also examined. This resulted in the elution time (tr) of FUR being very short while that of OLA being very long. Taking into account all the chromatographic behaviors of the analytes, the use of a triple solvent mixture MeOH–ACN–H_2_O NaH_2_PO4 20 mM at pH 3.6, 12:20:68 v/v/v was considered as the optimal solution. The system was isocratic (flow rate 1 mL min−1) for up to 23 min, in order to complete the elution of OLA. This was followed by a washing program (in 100% MeOH) for 5 min and the system was returned to the initial conditions (5 min). With the proposed method, the elution time of the first peak (FUR) was 10.8 min and of the last (OLA) 20.9 min, the pressure was low and the baseline stable.

Diluent composition in the final samples was also considered critical, as it affected the result (Table 2). For its selection, the signal intensity of the analytes (area under the curves, AUC), the shape of their chromatographic peaks, as well as the solvents to be used in the sample cleanup (urine) were taken into account. According to the results presented in Table 2, four solvents (water, methanol, ACN, and ISO) and their mixtures were tested. Some of them gave good chromatographic peaks and were subsequently used, as appropriate, in the urine cleanup techniques studied later. Finally, the ISO–ACN mixture 70:30 v/v at pH 5.4 was selected as optimal because it improves sample recoveries in the sample, although FUR AUC values were reduced by 20%.

**TABLE 2:** Effect of the diluent on method selectivity.

Diluent v/v	Blank	FLD intensity	Peak shape
ACN	Clean	Lower for PRP	FUR double peak
ACN–H_2_O 1:1	Clean	Lower for FUR	Satisfactory
H_2_O	Clean	Lower for all	Satisfactory
H_2_O FA 0.1%	Clean	No problem	Satisfactory
MeOH	Clean	Lower for PRP and FUR	FUR peak with fronting
MeOH FA 0.1%	Peak at tr_PRP_	Lower for PRP and FUR	FUR peak with fronting
MeOH–H_2_O 1:1	Clean	—	Satisfactory
NaH_2_PO_4_ pH 3.6	Peak at tr_PRP_	—	Satisfactory
MeOH–ACN 1:1	Clean	Lower for FUR	FUR double peak
MeOH–ISO 1:1	Clean	Lower for FUR	Satisfactory
ACN–ISO 1:1	Clean	Lower for FUR	Satisfactory
ACN–ISO–MEOH 1:1:1	Clean	Lower for FUR and higher for OLA	Satisfactory

Finally, the selection of appropriate wavelength pairs (λexc/λem) for the three analytes was carried out, based on their spectra in a Spectro RF‐5301PC fluorophotometer. For OLA, the 240/350 nm pair was chosen as optimal, since the compound gave a strong fluorescence signal. However, for PRP and FUR, various wavelengths were investigated (232/341, 286/341, 232/675 for PRP and 240/412, 270/412 and 327/412 nm for FUR). Their problem was that both active substances gave a strong signal in λex/λem pairs that were not selective, resulting in the appearance of many interferences. As an intermediate solution, the pairs 232 exc/675 em for PRP and 327exc/412em for FUR were chosen. In addition, a stepwise program of changing the two detector channels was used, according to Table S2.

### 3.2 Method Validation

For the reliable determination of the three analytes, an HPLC‐FLD method was developed and validated based on ICH Q2(R2), using standard solutions both in diluent and urine.

#### 3.2.1 System Suitability

The suitability of the chromatographic system was evaluated based on characteristic parameters that determine its integrity and efficiency (Table 3).

**TABLE 3:** System suitability parameters.

Analytes	Tr	Tf	k′	Rs	N	HETP, × 10^3^ USP
FUR	10.8	1.26	1.97	—	6626	37.72
PRP	13.2	1.21	2.59	4.36	7598	32.9
OLA	20.9	0.97	4.67	0.57	6959	35.92

Abbreviations: Tr: retention time, Tf: tailing factor, k′: capacity, Rs: resolution, N: number of theoretical plates, HETP: height equivalent of a theoretical plate.

#### 3.2.2 Selectivity

**FIGURE 1:**
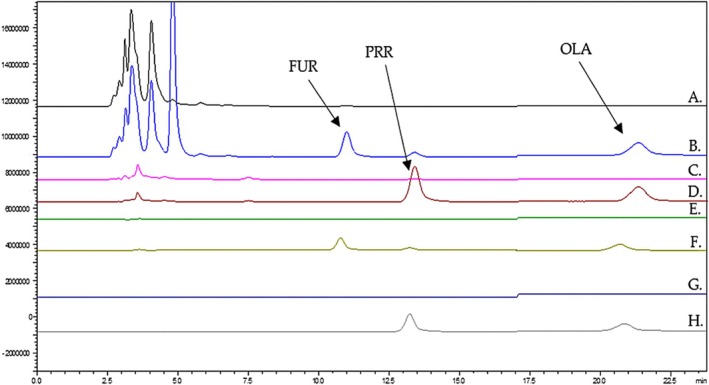
Typical chromatograms of A. blank of urine subjected to SPE (channel 1), B. Spiked standard solution of PRR 5 ng mL^−1^, FUR 5 ng mL^−1^ and OLA 10 ng mL^−1^ in urine subjected to SPE (channel 1), C. blank of urine subjected to SPE, (channel 2), D. Spiked standard solution in urine subjected to SPE (channel 2), E. blank with diluent (channel 1), F. standard solution of PRR 2 ng mL^−1^, FUR 2 ng mL^−1^ and OLA 4 ng mL^−1^ (channel 1), G. blank with diluent (channel 2), H. standard solution of the three analytes (channel 2).

The selectivity of the method was verified by comparing chromatograms obtained from a blank diluent and urine sample with the corresponding samples spiked with the three analytes. The HPLC method demonstrated selectivity, as there was no interference from substrate contaminations at the same retention times as the active substances (Figure 1). Carryover was evaluated by three successive injections of the highest calibration standard followed by a blank sample injection. No detectable peaks were observed in the blank chromatogram, confirming the absence of carryover.

#### 3.2.3 Linearity, LOD, and LOQ

The proposed chromatographic method was tested on two series of standard solutions in diluent and processed urine samples. Their linearity was evaluated at six concentration levels, with three replicates performed for each sample. Based on the AUC values obtained, calibration curves were constructed and their statistical characteristics are summarized in Table 4. The concentration levels were selected based on the expected urinary concentrations of the analytes, while also aiming to broaden the range as much as possible in order to account for potential metabolic variations and to ensure the method's applicability as a tool for personalized therapy. The expected concentrations are 13.7 ng/mL for FUR (Thakur et al. 2023), 25.2 ng/mL for PRP (Magiera et al. 2012), while for OLA there is no official data. For OLA the range was based on other similar, OLA related, studies in urine (Ottria et al. 2019). The similar values of the X variables in the two equations and the low intercept values ensure the effectiveness of the proposed extraction method. The reliability of the results was further confirmed by evaluating the % y‐intercept values (intercept × 100/100% response), which should be < 2%.

**TABLE 4:** Linear regression analysis data.

APIs	Concentration (ng mL^−1^)	Equation	%y intercept	*R* ^2^	LOD	LOQ
	Standard solutions in the diluent					
FUR	1–100	y = (306316.3 ± 866)x − 26,898 ± 40,318	0.1	0.999	0.4	1.3
PRP	1–100	y = (463859.9 ± 2091)x − 153,106 ± 96,987	0.5	0.999	0.7	2.1
OLA	2–200	y = (173675.1 ± 459)x − 122,316 ± 42,354	1.0	0.999	0.8	2.4
	Standard solutions spiked in urine					
FUR	1–100	y = (300,757.9 ± 2561)x + 296,799 ± 118,727	1.0	0.999	`1.3	3.9
PRP	1–100	y = (49,187.3 ± 3387)x − 102,076 ± 157,093	0.4	0.999	1.0	3.1
OLA	2–200	y = (195,437.3 ± 1245)x − 163,789 ± 115,471	0.4	0.999	1.9	5.9

The limits of detection (LOD) and quantification (LOQ) were determined using the following equations (Kamaris et al. 2025):

LOQ=10×Sy/x/Slope

LOD=3×Sy/x/Slope

where Sy/x is the residual standard deviation and Slope is the (x) variable of the calibration curve.

The low LOD, LOQ values for the three analytes highlight the sensitivity of the analytical method since they are comparable to those obtained with an HPLC‐MS/MS apparatus.

The matrix effect (ME) was evaluated by dividing the slope of the calibration curve obtained from standard solutions by the slope of the calibration curve generated from corresponding matrix samples (Aslan Akyol and Gokbulut 2026). The %ME values were 101.8% for FUR, 94.3% for PRP and 88.9% for OLA which are within the acceptable limits (80%–120%) (Mu et al. 2025).

#### 3.2.4 Precision and Repeatability

To evaluate the intra‐day (repeatability) and inter‐day (intermediate) precision, triplicate analyses were performed within one day and over three consecutive days, at three sample concentration levels (low, medium, high). Table 5 presents their results, expressed as %RSD values.

**TABLE 5:** Results for intraday and interday precision.

APIs	Repeatability		Intermediate precision				
	Concentration (ng mL^−1^)	RSD% (*n* = 3)		RSD% (*n* = 3)			Total (RSD%)
				First day	Second day	Third day	
	Standard solutions in the diluent						
FUR	1	0.4		0.4	0.7	1.7	1.7
	10	0.5		0.5	1.8	1.4	1.2
	100	0.7		0.7	1.3	0.4	0.9
PRP	1	0.6		0.6	0.8	0.6	1.5
	10	0.9		0.9	1.1	0.7	0.9
	100	1.3		1.3	0.2	0.5	0.4
OLA	2	0.9		0.9	1.1	2.1	1.9
	20	1.4		1.4	0.9	0.5	1.7
	200	1.3		1.3	1.7	0.6	1.1
	Standard solutions spiked in urine						
FUR	1	9.8		9.8	3.4	4.2	6.6
	10	4.4		4.4	6.0	4.0	7.2
	100	9.7		9.7	5.4	3.2	9.6
PRP	1	9.4		9.4	6.5	11.2	6.5
	10	1.9		1.9	2.8	2.6	10.9
	100	3.4		3.4	4.4	1.6	3.6
OLA	2	7.4		7.4	7.6	9.4	7.8
	20	2.0		2.0	4.0	6.5	9.4
	200	3.8		3.8	3.5	3.9	3.6

#### 3.2.5 Accuracy

To validate the accuracy, six samples of different concentrations for FUR, PRP, and OLA in both diluent and urine were prepared and analyzed. Their values were calculated based on their calibration curve equation and expressed as %Recovery. The accuracy of the method was adequate, as the mean %Recovery values were 100.1–100.6% (%RSD < 2.2) for the diluent samples and 98.2–100.6% (RSD < 9.1) for the corresponding urine samples (Table 6).

**TABLE 6:** %Recovery values of FUR, PRP, and OLA in the diluent and urine.

Concentration FUR (ng mL^−1^)	Found FUR (ng mL^−1^)	Recovery (%)	Concentration PRP (ng mL^−1^)	Found PRP (ng mL^−1^)	Recovery (%)	Concentration OLA (ng mL^−1^)	Found OLA (ng mL^−1^)	Recovery (%)
Standard solutions in the diluent								
1.0	1.03	102.8	1.0	1.03	103.0	2.0	2.1	102.9
5.0	4.9	97.7	5.0	5.0	99.1	10.0	10.1	101.2
10.0	10.3	102.5	10.0	10.0	99.6	20.0	19.4	97.0
25.0	24.9	99.5	25.0	24.8	99.3	50.0	49.7	99.4
100.0	100.6	100.6	100.0	100.0	100.0	200.0	99.9	99.9
% mean recovery		100.6	% mean recovery		100.2	% mean recovery		100.1
%RSD		2.1	%RSD		1.6	%RSD		2.2
Standard solutions spiked in urine								
1.0	0.9	86.7	1.0	1.1	112.5	2.0	2.3	114.0
5.0	5.4	108.3	5.0	4.5	89.1	10.0	8.8	88.5
10.0	9.8	98.3	10.0	9.9	98.8	20.0	19.6	98.2
25.0	24.3	97.3	25.0	25.6	102.3	50.0	51.2	102.5
100.0	100.1	100.1	100.0	99.9	99.9	200.0	199.8	99.9
% mean recovery		98.2	% mean recovery		100.5	% mean recovery		100.6
%RSD		7.9	%RSD		8.3	%RSD		9.1

#### 3.2.6 Robustness

To examine the robustness of the proposed method, the impact of minor changes to the operating conditions was assessed in terms of the %RSD values of the Tf and the peak area, AUC (Table 7).

**TABLE 7:** Robustness test.

Parameters	%RSD					
	FUR		PRP		OLA	
	AUC	Tf	AUC	Tf	AUC	Tf
Flow rate (±0.05 mL min^−1^)	1.0	0.4	0.6	1.1	1.4	1.8
Temperature (±2°C)	1.0	1.7	0.5	1.3	0.3	1.1
Mobile phase (±1%), B + C	1.8	1.0	2.0	1.9	0.4	1.3
Injection volume (±1 μL)	1.6	0.2	1.4	0.3	1.5	0.2
λmax (±1 nm)	0.3	0.5	0.5	0.6	0.2	0.7

According to the % RSD values found, the method was robust for all the examined parameters.

### 3.3 Sample Pretreatment

For the quantitative determination of FUR, PRP, and OLA in urine, a series of clean‐up and selective recovery techniques were tested. The aim of the study was to find the appropriate one without interference in the chromatograms which at the same time provide quantitative and reproducible recovery of the active substances at low detection limits. For this purpose, the following techniques were tested:

#### 3.3.1 LLE

LLE is a separation method based on the partitioning of an analyte between two immiscible liquids (organic and aqueous layers). The organic solvent is added to the aqueous solution containing the desired compound and the two phases are stirred to achieve the quantitative transfer of the analyte to the organic layer (Pramanik et al. 2024). In the present conditions, the aqueous layer was the urine sample spiked with 50 ng mL^−1^ FUR, 50 ng mL^−1^ PRP and 100 ng mL^−1^ OLA, while as organic extraction solvents were tested dichloromethane, ethyl acetate, chloroform and diethyl ether. More specifically, to 1.0 mL of sample, adjusted to pH 3 with 0.1 mL of 350 mM NaH_2_PO_4_ buffer (so that FUR was in its non‐ionized form) 1.0 mL of organic extraction solvent was added. The sample was vortexed (15 s) and after being left to separate the two layers, exactly 0.8 mL of the organic solvent was obtained. The same process of adding the organic solvent was repeated two more times and after the organic layers were collected, they were evaporated gently using nitrogen gas. Among the four solvents tested, dichloromethane was the most efficient, but although its selectivity, the recoveries of the three analytes were 32% for PRP, 46% for OLA and only 2% for FUR. Similar problems were encountered using ethyl acetate (low recoveries) and chloroform (zero recovery for OLA and PRP). Finally, diethyl ether gave blank samples with interferences at the same tr as FUR and OLA. A variant of the LLE method, the Dispersion Liquid–Liquid Microextraction (DLLE) procedure, which has been reported to be used for the isolation of FUR, was then applied. (Han et al. 2013).

According to the method, 1.0 mL of MeOH or ACN containing 50 μL of dichloromethane was added, by insufflation, to 5 mL of spiked urine (concentration 50 ng mL^−1^ FUR, 50 ng mL^−1^ PRP, 100 ng mL^−1^ OLA). The rapid influx of the organic solvent and the sonication (5 min) of the sample led to the formation of a cloudy solution. After centrifugation (10 min, 5000 rpm), a dichloromethane precipitate was formed from which 30 μL was collected. This amount was evaporated with nitrogen gas, reconstituted with 1 mL of MeOH‐H_2_O 1:1 v/v, and analyzed. According to the results, the recoveries of the active ingredients were < 5%, and for this reason, the DLLE was rejected. In practice, the method appears to present problems with both low recoveries and the inability to collect 30 μL of dichloromethane from a sample with a total volume of 6 mL (5 mL of urine +1 mL of MeOH).

#### 3.3.2 Protein Precipitation With Acids or Organic Solvents

Protein precipitation is a well‐established technique for preparing samples for HPLC analysis, as it removes protein matrix interferences and enables reliable determination of small molecules. The most commonly used precipitating agents are acidic reagents and organic solvents (Salina and Regazzoni 2024).

Perchloric acid (PCA) and trifluoroacetic acid (TFA) were used as acidic reagents in this experiment. Specifically, 10 μL of ice‐cold 4% PCA solution were added to a cold sample (500 μL) of spiked urine (50 ng mL^−1^ FUR, 50 ng mL^−1^ PRP, 100 ng mL^−1^ OLA). The sample was mixed in an ice bath (15 min) and placed in the freezer (15 min). Centrifugation (5000 rpm, 30 min) was then followed and 200 μL of the supernatant, after being neutralized with 5 μL of 0.5 M KOH (Viode et al. 2023), were analyzed by HPLC. The purification was considered insufficient. More specifically, along with the solvent front, a high intensity and thick substrate peak was eluted, which extended up to the elution time of FUR, while corresponding interferences (peaks) were also observed in PRP and OLA retention times. Alternatively, an attempt was made to precipitate the proteins with 1% TFA (Hagino et al. 2025). The results were better, as the contamination problems were mainly located in FUR which could not be quantified, while the recovery rates of PRP and OLA were 69% and 58%, respectively. In an attempt to further clean up the sample by applying a SPE technique, after protein acid precipitation, the recovery rates of the APIs were 60% for FUR, 64% for PRP and 56% for OLA.

Alternatively, two organic solvents, methanol and ACN, were tested for protein precipitation. Specifically, a specific volume of organic solvent (methanol or ACN) was added to 500 μL of spiked urine (50 ng mL^−1^ FUR, 50 ng mL^−1^ PRP, 100 ng mL^−1^ OLA) in ratios of 1:1 and 1:3 v/v. After the sample was mixed with a vortex (5 min), it remained for 30 min at a temperature of −18°C, centrifuged (10,000 rpm, 5 min), and the supernatant was analyzed by HPLC. In addition to the disadvantage that a large volume of organic solvent is required to achieve precipitation, the main problem was the lack of purity of the samples. Between the two solvents, methanol (in a ratio of 1:3 v/v) was more effective. However, additional sample purification was performed using the “freezing out” technique. Thus, the samples after methanol precipitation were placed in the freezer for 10 min to 2 h. In none of these individual cases did the results significantly improve, as the best recovery rates were 80% for PRP, 70% for OLA, while FUR was impossible to quantify.

#### 3.3.3 Salting‐Out Technique

The salting‐out technique takes place at high salt concentrations which reduces the solubility of proteins, causing their precipitation. Thus, the supernatant aqueous solution can be used for the analysis of smaller molecules without matrix interference (De Araújo Júnior et al. 2019). For this purpose, in the present experimental conditions, 2 g NaCl were added to 1 mL of spiked urine samples (50 ng mL^−1^ FUR, 50 ng mL^−1^ PRP, 100 ng mL^−1^ OLA), vortexed for 5 min and centrifuged (6000 rpm for 15 min). The supernatant was collected and analyzed by HPLC (% Recoveries were 92% for FUR, 89% for PRP and 70% for OLA). Similar % recoveries were found when the desalting technique was followed by an SPE extraction method.

As an alternative, a combination of a salting‐out and LLE technique was used (SALLE). SALLE can separate water‐miscible organic solvents such as ACN from plasma or other aqueous biofluids, extracting a wide range of drugs (Tang and Weng 2013). For this purpose, 2 g NaCl were added to 1.0 mL of spiked and unspiked urine and centrifuged (15 min 6000 rpm). 0.8 mL of the supernatant were collected and after the addition of 0.1 mL buffer solution (NaH_2_PO_4_ buffer 350 mM at pH 3) were extracted with 0.8 mL of ACN (%Recoveries were 66% for FUR, 98% for PRP and 91% for OLA). The same procedure was repeated using dichloromethane (three times) instead of ACN. Based on the results, the method was not considered adequate since the %Recoveries values were 27% for PRP, 38% for OLA, while for FUR it was below 1%.

#### 3.3.4 SPE

One of the most widely used techniques for the purification and pretreatment of biological samples is SPE, as it offers high selectivity, reproducibility, and automation. It is based on the selective retention of analytes on a solid adsorbent material, allowing the removal of interfering matrix components (Mandal et al. 2023).

In the present experimental conditions, a preliminary study was carried out in order to establish the optimal interaction mechanism between the analytes and the solid adsorbent. Thus, a series of cartridges with different chemical structures and the same technical characteristics (C18, C8, phenyl and CN Supelco Supelclean‐SPE, 3 mL) were tested. In all cases, the SPE procedure included the following steps: each cartridge was conditioned with 2 mL MeOH, 1 mL H_2_O and 1 mL of 0.5% FA. Then, it was loaded with 1 mL of spiked urine (with 50 ng mL^−1^ FUR, 50 ng mL^−1^ PRP, 100 ng mL^−1^ OLA, adjusted to pH 3.6 with 50 μL NaH_2_PO_4_ 350 mM) and washed with 1 mL of 0.5% FA. For the elution of the samples, a 1 mL mixture of three solvents (MeOH‐ACN‐ISO 1:1:1 v/v/v) was used, which had a high dissolving capacity and elution power (Lee et al. 2007). In order to control the process at all stages, samples were collected and analyzed from both the eluate and the cartridge wash. According to the results (Table 8), the C18 solid adsorbent was chosen as the optimal one, as the eluate was cleaner and gave the best API recoveries.

**TABLE 8:** %Recovery values using different types of cartridges.

Cartridge	%Recovery FUR	%Recovery PRP	%Recovery OLA
C18‐Supelclean 3 mL	91	93	89
C8‐Supelclean 3 mL	89	87	90
CN‐Supelclean 3 mL	63	54	57
Phe‐Supelclean 3 mL	62	158	78
C18‐Supelclean 6 mL	85	77	69
C18‐Empore single	103	77	70
C18‐Empore double	65	76	74

Subsequently, four cartridges of the same chemical structure (C18) but with different technical characteristics were tested and evaluated (Supelco Supelclean ‐SPE 3 mL tubes, Supelco Supelclean ‐SPE 6 mL tubes, Empore C18‐SD single‐layer, Empore C18‐SD double‐layer). Completing the investigation, the Supelco Supelclean ‐SPE 3 mL cartridge was selected as the most suitable for performing further optimization experiments (Table 8).

The factors that also had to be studied concerned parameters that affect the extraction process such as pH and the ratio of solvents in the elution mixture. Because such a system is multifactorial, an experimental design methodology was applied (Design Expert Version 11 software, Stat‐Ease Inc., Minneapolis, MN) using a Mixture Crossed D‐optimal (CDO) model. The pH was investigated at values ranging from 3 to 10 (adjustment was made by adding 100 μL of 350 mM NaH_2_PO_4_ solution), while the total volume of MeOH‐ACN and ISO was set at 1.0 mL (Table 9).

**TABLE 9:** Description of the D‐optimal experimental design model.

Study type	Combined		Subtype	Randomized
Design type	D‐optimal		Runs	28
Design model	Quadratic × Quadratic		Blocks	No blocks
Mixture	Components	A (MeOH) + B (ACN) + C (isopropanol) = 1 mL		
Process	Factors	D pH_elution_ = 3–10^a^		
	Responses	%Recovery of FUR, PRP, and OLA		

^a^ The reported pH refers to the phosphate buffer (NaH_2_PO_4_) added at a 1:10 (v/v) ratio to the organic solvent during elution.

Based on the best fit of the data (D‐optimal algorithm), the model suggested conducting 28 experiments (Table S3), the results of which were statistically processed and adjusted for all possible correlation combinations. Each experiment was conducted in triplicate. Thus, a quadratic order was used for the “mixture components” which was crossed with a quadratic order of the pH factor. Similarly, for each response (% Recovery) a separate processing model was selected based on its significance criterion, i.e., the p‐value should be < 0.05 (Table 10). It is also desirable that the standard deviation, defined as the “Square Root of Variance”, should be as small as possible, while the F‐value, which expresses the ratio of systematic dispersion to random unsystematic, should be > 1. The goal is for the “R‐squared” and “adjusted R‐squared” to be as close to unity as possible, so that 100% of the observed variance can be explained by the model. Finally, the term “Sufficient Accuracy” expresses the signal‐to‐noise ratio and is preferred to have values > 4.

**TABLE 10:** Combined model fit summary.

Parameters	FUR	PRP	OLA
SD	9.88	7.69	5.71
CV%	13.76	9.7	7.51
*R* ^2^	0.89	0.88	0.97
Adjusted *R* ^2^	0.88	0.86	0.96
Predicted *R* ^2^	0.84	0.83	0.92
Adeq precision	18.52	17.46	30.36
Mixture *p* value Process *p* value	(Q×M) < 0.0001	(Q×M) < 0.0001	(Q×Q) < 0.0001
	—	—	(Q×Q) < 0.0016

**FIGURE 2:**
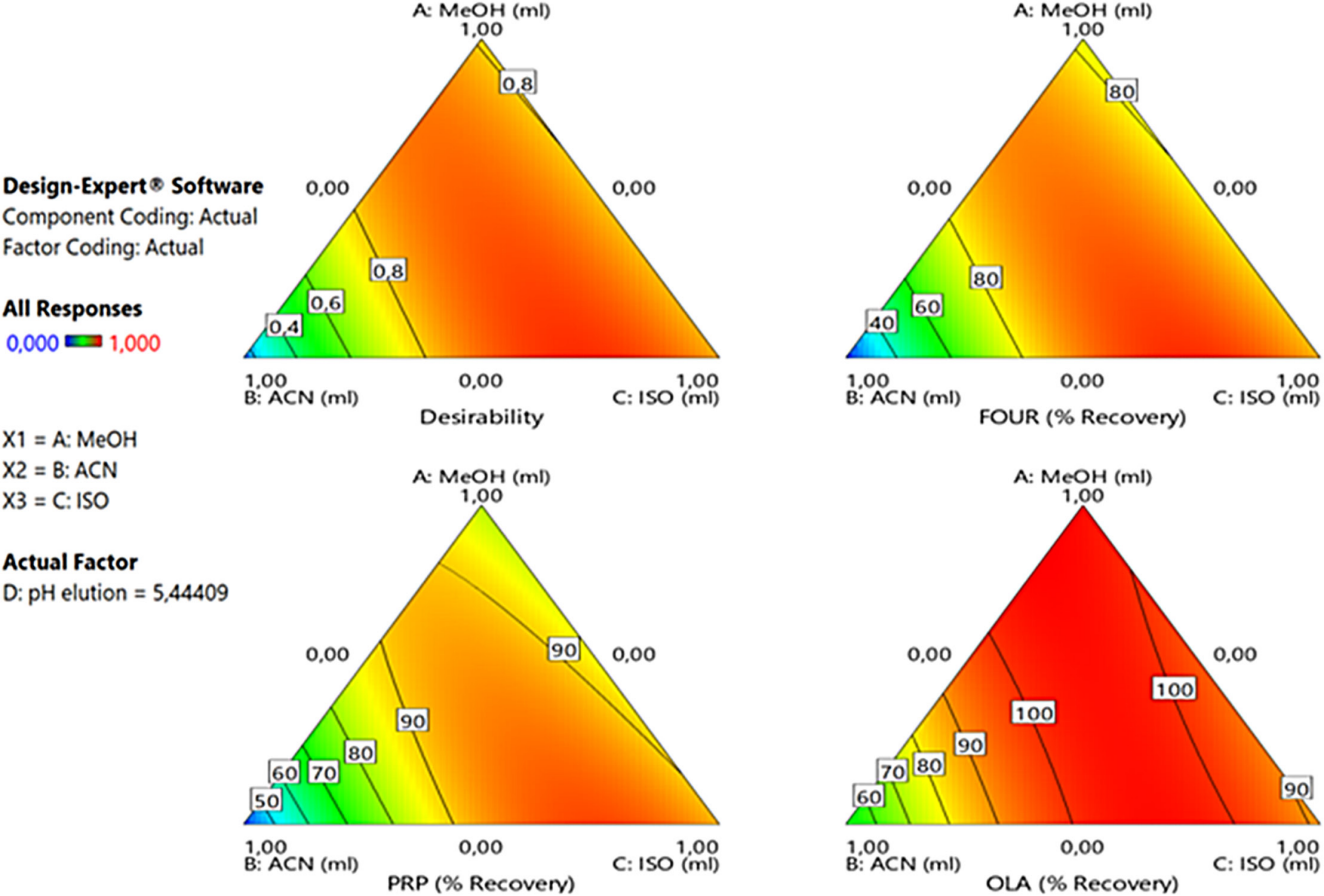
Isometric curve diagrams of the percentage ratio of the mobile phase components for optimal recovery values of FUR, PRP, and OLA.

**FIGURE 3:**
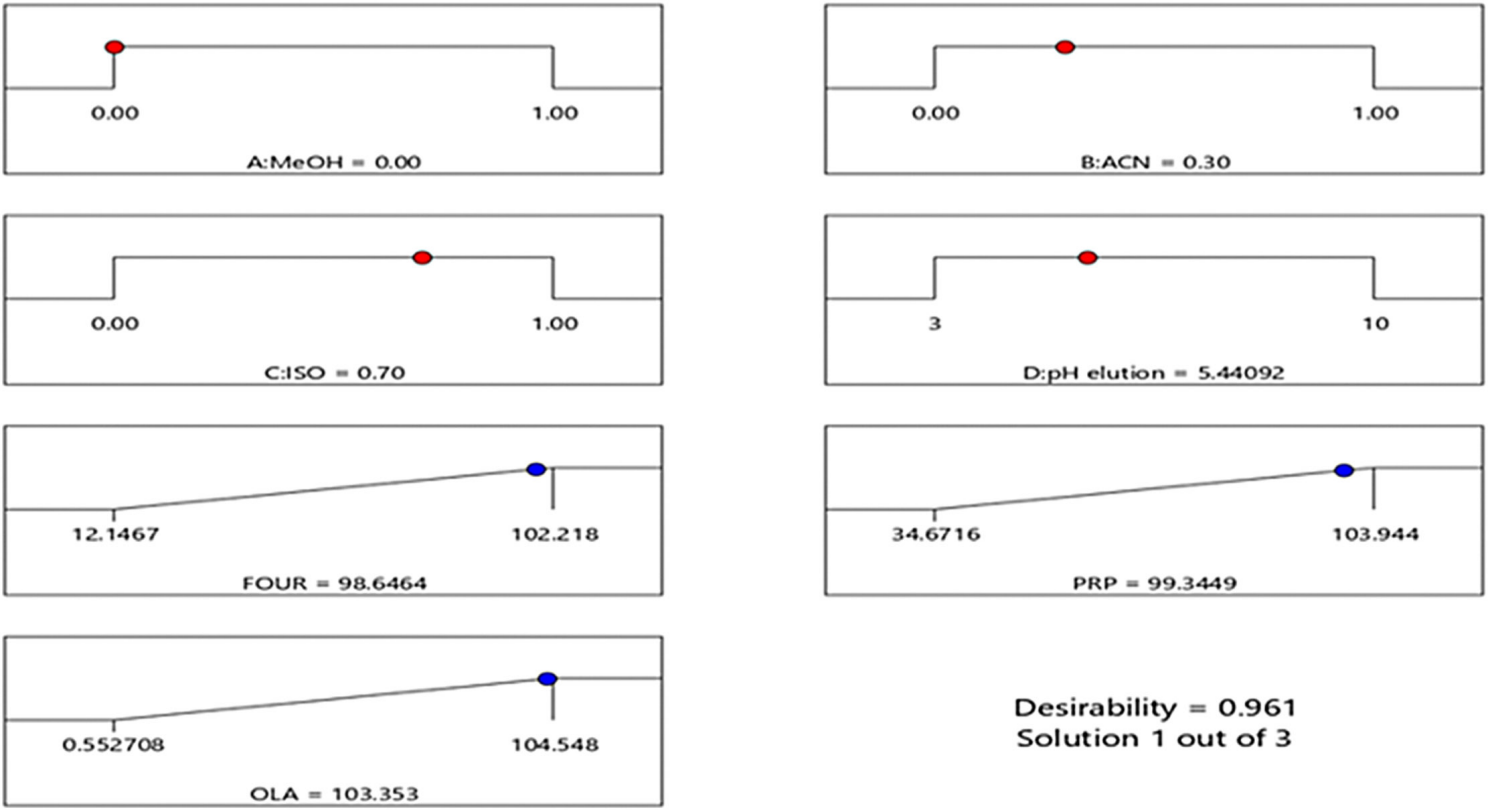
Diagrams depicting the optimal suggested values for the factors and the expected values of the responses.

The significance of the factors was assessed using analysis of variance (ANOVA). For a significant level of 95% (α = 0.05), those with *p* < 0.05 were considered worthy of study (Table S4). Therefore, the %Recovery values for FUR and PRP seem to be affected only by the MeOH‐ACN‐ISO mixture, while for OLA the effect of pH was equally significant. The design of isometric curves also contributed to a better understanding of the effects of the solvent mixture on the responses (Figure 2), when the pH has a specific value. In Figure 3 it is seen that the best recovery values (red area) are achieved by increasing ISO. It also showed that methanol does not favor FUR and PRP recoveries.

The polynomial equations that mathematically correlate the selected responses are presented in Table S5, while the predicted vs. actual values of FUR, PRP, OLA were illustrated at Figure S4. To find the optimal solution of the model, Derringer's “Desirability Function” was used (Bourguignon and Massart 1991). The function suggested 3 possible solutions with different desirability values, of which the optimal one is presented in Figure 3 and indicates as a suitable eluent the ISO‐ACN mixture 70:30 v/v at pH 5.4.

To verify the results, the proposed extraction procedure was repeated on 5 spiked urine samples (10 ng mL^−1^ FUR, 10 ng mL^−1^ PRP, 20 ng mL^−1^ OLA) where the mean % Recovery value was for FUR 95.6 (%RSD = 5.6) for PRP 102.8 (%RSD = 4.2) and for OLA 95.5 (%RSD = 4.8).

### 3.4 Green, Practical, and Performance Assessment

#### 3.4.1 Modified Green Analytical Procedure Index (MoGAPI) Assessment Tool

**FIGURE 4:**
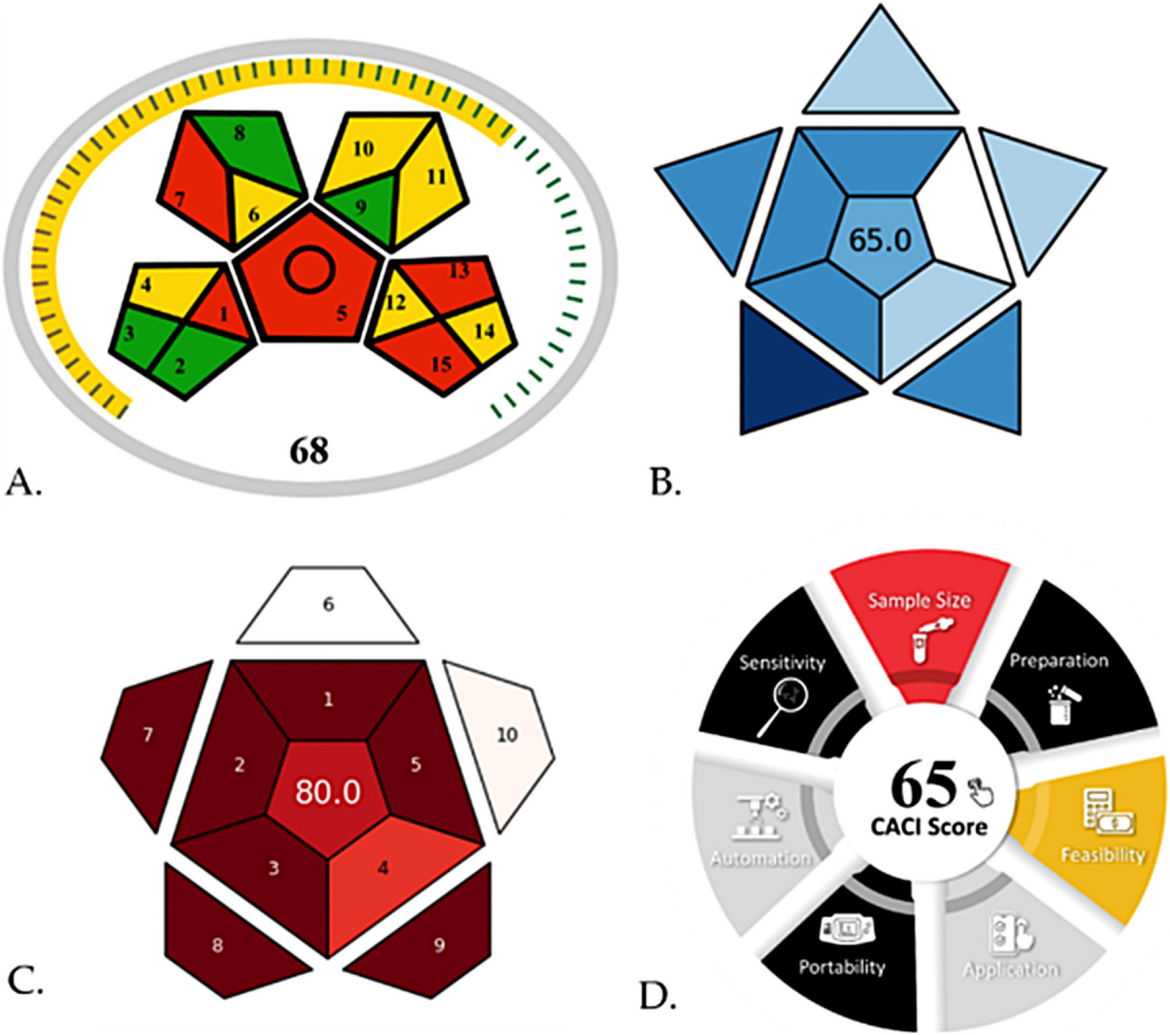
Green practical and performance evaluation of the proposed analytical method based on the indicators: A) MoGAPI B) BAGI C) RAPI and D) CACI.

As an extension of the GAPI index, MoGAPI accurately evaluates analytical methods in terms of environmental impact and safety, using pentagonal pictorial representations with color coding. To properly assess the environmental friendliness of the method, the sample pretreatment method, the consumption of reagents and solvents, and the equipment are taken into account. A MoGapi score of 68 indicates an environmentally friendly analytical method, with reduced chemical consumption and waste generation (Mansour et al. 2024) (Figure 4A).

#### 3.4.2 Blue Applicability Grade Index (BAGI) Assessment Tool

BAGI is a complementary tool to Green Assessment methods designed to assess the applicability of an analytical method. It considers ten key characteristics related to the practical aspects of White Analytical Chemistry (WAC). The method's compliance with each criterion is visually represented using an asterisk pictogram, where dark blue, blue, light blue, and white indicate high, moderate, low, and no compliance, respectively. In this assessment, the method achieved a BAGI score of 65, indicating that the protocol has strong applicability potential (Manousi et al. 2023) (Figure 4B).

#### 3.4.3 Red Analytical Performance Index (RAPI) Assessment Tool

The RAPI tool was utilized to assess and visualize the overall performance of an analytical method, focusing on efficiency, reliability, and suitability for real‐time analysis rather than exclusively on environmental or green aspects. Like BAGI, RAPI is developed within the framework of WAC and linked to the red components of an asteroid pictogram with darker shades of red indicating high performance. Key factors include selectivity, sensitivity, accuracy, and precision. It also considers detection limits, analysis time, complexity of sample preparation, instrumentation requirements, and overall reproducibility. The proposed method received a RAPI score of 80, which demonstrates strong performance (Nowak et al. 2025) (Figure 4C).

#### 3.4.4 Analytical Chemistry Index (CACI) Assessment Tool

CACI is a new assessment tool that evaluates analytical methods based on the fundamental traits of click chemistry: simplicity, efficiency, and reliability. Click chemistry encourages the design of analytical procedures that not only exhibit high‐quality performance levels but also reduce procedural complexity. From this perspective, factors such as sample size, preparation, feasibility, applicability, portability, sensitivity, and degree of automation are examined. The outcomes are displayed using a color‐coded pictogram, where colored sections represent high performance, gray indicates moderate performance, and black denotes poor performance or failure to meet the required standards. For the proposed method, a practical score of 65% is considered satisfactory (Mansour et al. 2025) (Figure 4D).

## 4 Conclusions

For the simultaneous determination of free FUR, PRP, and OLA in urine, a reversed phase HPLC method with an FLD detector is proposed. Critical points that ensured its high performance (sensitivity) were the isocratic elution of the mobile phase and the use of a gradually varying detection program (λ exc/em) in the two channels of the detector. To ensure the sufficient separation capacity of the chromatographic system, three solvents were used: (A) methanol, (B) ACN, and (C) 20 mM NaH_2_PO_4_ buffer pH 3.6 12:20:68 v/v/v. Accordingly, SPE (C 18, Supelclean‐SPE cartridges of 3 mL) was chosen for the sample cleanup, which was optimized by applying the D‐Optimal experimental design methodology. Based on the proposed model, the quantitative collection of analytes was achieved with a mixture of ISO–ACN 70:30 v/v at pH 5.4. Finally, the method was evaluated with specific statistical tools and was characterized as environmentally friendly and applicable.

## Funding

The authors have nothing to report.

## Supporting information


**Figure S1:** Chemical structure of OLA, PRP, and FUR.
**Table S1:** Physicochemical properties.
**Figure S2:** Chromatogram of FUR, PRP, and OLA using MeOH‐NaH_2_PO_4_ 20 mM, pH = 3.6 45:55 v/v, as the mobile phase (isocratic elution, flow rate 0.8 mL/min) and a Nucleosil Macherey‐Nagel C8 (250 mm × 4.6 mm, 5 μm), 50°C as stationary (black) and blank chromatogram (pink).
**Figure S3:** Chromatogram of FUR, PRP, and OLA using MeOH‐NaH_2_PO_4_ 20 mM, pH = 3.6, 45:55 v/v, as the mobile phase (gradient elution, flow rate 1 mL/min) and a Nucleosil Macherey‐Nagel C8 (250 mm × 4.6 mm, 5 μm), 45°C, as stationary (black) and blank chromatogram (pink), at HPLC‐UV instrumentation.
**Table S2:** Method FLD RF‐20A conditions.
**Table S3:** Sample processing conditions and %recovery results.
**Table S4:** Optimal fitting models for the selected responses (analysis of variance).
**Table S5:** Polynomial model equations for the selected responses.
**Figure S4:** Predicted vs. actual values of FUR, PRP, and OLA.

## References

[bmc70529-bib-0001] Alampanos, V. , and V. Samanidou . 2021. “An Overview of Sample Preparation Approaches Prior to Liquid Chromatography Methods for the Determination of Parabens in Biological Matrices.” Microchemical Journal 164: 105995. 10.1016/j.microc.2021.105995.

[bmc70529-bib-0002] Alothman, Z. A. , K. M. Alsheetan , H. Y. Aboul‐Enein , and I. Ali . 2020. “Applications of Shun Shell Column and Nanocomposite Sorbent for Analysis of Eleven Anti‐Hypertensive in Human Plasma.” Journal of Chromatography B 1146: 122125. 10.1016/j.jchromb.2020.122125. 32371329

[bmc70529-bib-0003] Aslan Akyol, B. , and C. Gokbulut . 2026. “A Simple and Sensitive Methodology for Quantification of Aflatoxins in Milk and Butter by HPLC: Method Development, Validation and Application to Real Samples.” Journal of Food Composition and Analysis 149: 108794. 10.1016/j.jfca.2025.108794.

[bmc70529-bib-0004] Badawy, M. E. I. , M. A. M. El‐Nouby , P. K. Kimani , L. W. Lim , and E. I. Rabea . 2022. “A Review of the Modern Principles and Applications of Solid‐Phase Extraction Techniques in Chromatographic Analysis.” Analytical Sciences 38, no. 12: 1457–1487. 10.1007/s44211-022-00190-8. 36198988 PMC9659506

[bmc70529-bib-0005] Baranowska, I. , P. Markowski , and J. Baranowski . 2006. “Simultaneous Determination of 11 Drugs Belonging to Four Different Groups in Human Urine Samples by Reversed‐Phase High‐Performance Liquid Chromatography Method.” Analytica Chimica Acta 570, no. 1: 46–58. 10.1016/j.aca.2006.04.002.

[bmc70529-bib-0006] Baranowska, I. , and J. Płonka . 2016. “Monitoring of Biogenic Amines and Drugs of Various Therapeutic Groups in Urine Samples With Use of HPLC.” Biomedical Chromatography 30, no. 4: 652–657. 10.1002/bmc.3614. 26362402

[bmc70529-bib-0007] Bourguignon, B. , and D. L. Massart . 1991. “Simultaneous optimization of Several Chromatographic Performance Goals Using Derringer's Desirability Function.” Journal of Chromatography A 586, no. 1: 11–20. 10.1016/0021-9673(91)80019-D.

[bmc70529-bib-0008] Bruin, M. A. C. , G. S. Sonke , J. H. Beijnen , and A. D. R. Huitema . 2022. “Pharmacokinetics and Pharmacodynamics of PARP Inhibitors in Oncology.” Clinical Pharmacokinetics 61, no. 12: 1649–1675. 10.1007/s40262-022-01167-6. 36219340 PMC9734231

[bmc70529-bib-0009] Catanese, L. , H. Rupprecht , T. B. Huber , et al. 2024. “Non‐Invasive Biomarkers for Diagnosis, Risk Prediction, and Therapy Guidance of Glomerular Kidney Diseases: A Comprehensive Review.” International Journal of Molecular Sciences 25, no. 6: 3519. 10.3390/ijms25063519. 38542491 PMC10970781

[bmc70529-bib-0010] Cavalu, S. , S. Saber , A. E. Amer , et al. 2024. “The Multifaceted Role of Beta‐Blockers in Overcoming Cancer Progression and Drug Resistance: Extending Beyond Cardiovascular Disorders.” FASEB Journal 38, no. 13: e23813. 10.1096/fj.202400725RR.38976162

[bmc70529-bib-0012] Chachlioutaki, K. , M. Liogka , P. M. Petinari , et al. 2025. “Electrospun Mucoadhesive Nanofibrous Films for Intranasal Delivery of Propranolol Hydrochloride for Migraine Prophylaxis.” Journal of Drug Delivery Science and Technology 114: 107552. 10.1016/j.jddst.2025.107552.

[bmc70529-bib-0013] Chen, X. , Q. Wen , L. Kou , X. Xie , J. Li , and Y. Li . 2023. “Incidence and Risk of Hypertension Associated With PARP Inhibitors in Cancer Patients: A Systematic Review and Meta‐Analysis.” BMC Cancer 23, no. 1: 107. 10.1186/s12885-023-10571-5. 36717798 PMC9887889

[bmc70529-bib-0014] Darwish, I. A. , and N. Y. Khalil . 2023. “Development and Comparative Evaluation of Two Different Label‐Free and Sensitive Fluorescence Platforms for Analysis of Olaparib: A Recently FDA‐Approved Drug for the Treatment of Ovarian and Breast Cancer.” Molecules 28, no. 18: 6524. 10.3390/molecules28186524. 37764300 PMC10537137

[bmc70529-bib-0015] Daumar, P. , R. Dufour , C. Dubois , F. Penault‐Llorca , M. Bamdad , and E. Mounetou . 2018. “Development and Validation of a High‐Performance Liquid Chromatography Method for the Quantitation of Intracellular PARP Inhibitor Olaparib in Cancer Cells.” Journal of Pharmaceutical and Biomedical Analysis 152: 74–80. 10.1016/j.jpba.2018.01.036. 29414021

[bmc70529-bib-0016] De Araújo Júnior, M. B. , P. D. A. Pessoa Filho , and E. A. Miranda . 2019. “Effect of the Initial Concentration on the Equilibrium Liquid Phase Concentration at Salting Out of Proteins.” Journal of Chemical Technology & Biotechnology 94, no. 11: 3706–3712. 10.1002/jctb.6176.

[bmc70529-bib-0017] Derwand, D. , S. Rzeppa , S. C. Voss , A. Zschiesche , and A. M. Keiler . 2024. “QuEChERS as Alternative Extraction Procedure in Doping Analyses.” Drug Testing and Analysis 16, no. 9: 936–941. 10.1002/dta.3610. 38043941

[bmc70529-bib-0018] Di Fulvio, M. , Y. D. Rathod , and S. Khader . 2025. “Diuretics: A Review of the Pharmacology and Effects on Glucose Homeostasis.” Frontiers in Pharmacology 16: 1513125. 10.3389/fphar.2025.1513125. 40223924 PMC11985539

[bmc70529-bib-0019] El‐Saharty, Y. S. 2003. “Simultaneous High‐Performance Liquid Chromatographic Assay of Furosemide and Propranolol HCL and Its Application in a Pharmacokinetic Study.” Journal of Pharmaceutical and Biomedical Analysis 33, no. 4: 699–709. 10.1016/S0731-7085(03)00229-2. 14623596

[bmc70529-bib-0020] Ferrer, F. , P. Tetu , L. Dousset , et al. 2024. “Tyrosine Kinase Inhibitors in Cancers: Treatment Optimization – Part II.” Critical Reviews in Oncology/Hematology 200: 104385. 10.1016/j.critrevonc.2024.104385. 38810843

[bmc70529-bib-0021] Galaon, T. , S. Udrescu , I. Sora , V. David , and A. Medvedovici . 2007. “High‐Throughput Liquid‐Chromatography Method With Fluorescence Detection for Reciprocal Determination of Furosemide or Norfloxacin in Human Plasma.” Biomedical Chromatography 21, no. 1: 40–47. 10.1002/bmc.715. 17080503

[bmc70529-bib-0022] Gomez, G. C. , C. V. Plessing R , C. G. Godoy M , R. Reinbach H , and R. Godoy R . 2005. “Method Validation for the Determination of Furosemide in Plasma by Liquid‐Liquid Extraction and High‐Performance Liquid Chromatography With Fluorescence Detection.” Journal of the Chilean Chemical Society 50, no. 2: 479–482. 10.4067/S0717-97072005000200008.

[bmc70529-bib-0023] Gui, J. 2025. “Analysis of Global Ovarian Cancer Disease Burden and Its Changing Trend From 1990 to 2021.” BMC Women's Health 25, no. 1: 352. 10.1186/s12905-025-03904-y. 40671068 PMC12265128

[bmc70529-bib-0024] Gulsun, T. , H. Demir , S. Ebadi Borna , and S. Sahin . 2025. “Furosemide Nanosuspensions: Preparation, Characterization and Evaluation of the Interplay Between Solubility and Permeability.” Journal of Drug Delivery Science and Technology 112: 107302. 10.1016/j.jddst.2025.107302.

[bmc70529-bib-0025] Hagino, K. , K. Masuda , Y. Shimizu , and N. Ichihashi . 2025. “Sustainable Regeneration of 20 Aminoacyl‐tRNA Synthetases in a Reconstituted System Toward Self‐Synthesizing Artificial Systems.” Science Advances 11, no. 14: eadt6269. 10.1126/sciadv.adt6269. 40173221 PMC11963985

[bmc70529-bib-0026] Han, D. , B. Tang , and K. H. Row . 2013. “Determination of Diuretic Drugs in Human Urine Using Dispersive Liquid–Liquid Microextraction by High Performance Liquid Chromatography.” Journal of Liquid Chromatography & Related Technologies 36, no. 15: 2069–2081. 10.1080/10826076.2012.712931.

[bmc70529-bib-0027] Hedera, P. , F. Cibulčík , and T. L. Davis . 2013. “Pharmacotherapy of Essential Tremor.” Journal of Central Nervous System Disease 5: JCNSD.S6561–JCNSD.S6555. 10.4137/JCNSD.S6561. PMC3873223 24385718

[bmc70529-bib-0028] Huang, X. , E. Dorhout Mees , P. Vos , S. Hamza , and B. Braam . 2016. “Everything We Always Wanted to Know About Furosemide but Were Afraid to Ask.” American Journal of Physiology. Renal Physiology 310, no. 10: F958–F971. 10.1152/ajprenal.00476.2015. 26911852

[bmc70529-bib-0031] Kamaris, G. , N. Pantoudi , and C. K. Markopoulou . 2025. “Development and Validation of HPLC‐DAD/FLD Methods for the Determination of Vitamins B1, B2, and B6 in Pharmaceutical Gummies and Gastrointestinal Fluids—In Vitro Digestion Studies in Different Nutritional Habits.” Molecules (Basel, Switzerland) 30, no. 19: 3902. 10.3390/molecules30193902. 41097322 PMC12525618

[bmc70529-bib-0032] Kim, H. K. , J. H. Hong , M. S. Park , J. S. Kang , and M. H. Lee . 2001. “Determination of Propranolol Concentration in Small Volume of Rat Plasma by HPLC With Fluorometric Detection.” Biomedical Chromatography 15, no. 8: 539–545. 10.1002/bmc.110. 11748690

[bmc70529-bib-0033] Knox, C. , M. Wilson , C. M. Klinger , et al. 2024. “DrugBank 6.0: The DrugBank Knowledgebase for 2024.” Nucleic Acids Research 52, no. D1: D1265–D1275. 10.1093/nar/gkad976. 37953279 PMC10767804

[bmc70529-bib-0034] Lanser, D. A. C. , M. B. A. Van Der Kleij , G. D. M. Veerman , et al. 2023. “Design and statistics of pharmacokinetic drug‐drug, herb‐drug, and food‐drug interaction studies in oncology patients.” Biomedicine & Pharmacotherapy 163: 114823. 10.1016/j.biopha.2023.114823. 37172331

[bmc70529-bib-0035] Lapini, A. , O. Caffo , G. N. Conti , et al. 2023. “Matching BRCA and Prostate Cancer in a Public Health System: Report of the Italian Society for Uro‐Oncology (SIUrO) Consensus Project.” Critical Reviews in Oncology/Hematology 184: 103959. 10.1016/j.critrevonc.2023.103959. 36921782

[bmc70529-bib-0036] Lee, H.‐B. , K. Sarafin , and T. E. Peart . 2007. “Determination of β‐Blockers and β2‐Agonists in Sewage by Solid‐Phase Extraction and Liquid Chromatography–Tandem Mass Spectrometry.” Journal of Chromatography A 1148, no. 2: 158–167. 10.1016/j.chroma.2007.03.024. 17408682

[bmc70529-bib-0037] Li, S. , V. Silvestri , G. Leslie , et al. 2022. “Cancer Risks Associated With *BRCA1* and *BRCA2* Pathogenic Variants.” Journal of Clinical Oncology 40, no. 14: 1529–1541. 10.1200/JCO.21.02112. 35077220 PMC9084432

[bmc70529-bib-0038] Luketic, V. A. , and A. J. Sanyal . 2000. “Esophageal Varices.” Gastroenterology Clinics of North America 29, no. 2: 337–385. 10.1016/S0889-8553(05)70119-9. 10836186

[bmc70529-bib-0039] Magiera, S. , I. Baranowska , and J. Kusa . 2012. “Development and Validation of UHPLC–ESI‐MS/MS Method for the Determination of Selected Cardiovascular Drugs, Polyphenols and Their Metabolites in Human Urine.” Talanta 89: 47–56. 10.1016/j.talanta.2011.11.055. 22284458

[bmc70529-bib-0040] Mandal, S. , R. Poi , D. K. Hazra , I. Ansary , S. Bhattacharyya , and R. Karmakar . 2023. “Review of Extraction and Detection Techniques for the Analysis of Pesticide Residues in Fruits to Evaluate Food Safety and Make Legislative Decisions: Challenges and Anticipations.” Journal of Chromatography B 1215: 123587. 10.1016/j.jchromb.2022.123587. 36628882

[bmc70529-bib-0041] Manousi, N. , W. Wojnowski , J. Płotka‐Wasylka , and V. Samanidou . 2023. “Blue Applicability Grade Index (BAGI) and Software: A New Tool for the Evaluation of Method Practicality.” Green Chemistry 25, no. 19: 7598–7604. 10.1039/D3GC02347H.

[bmc70529-bib-0042] Mansour, F. R. , A. Bedair , and M. Locatelli . 2025. “Click Analytical Chemistry Index as a Novel Concept and Framework, Supported With Open Source Software to Assess Analytical Methods.” Advances in Sample Preparation 14: 100164. 10.1016/j.sampre.2025.100164.

[bmc70529-bib-0043] Mansour, F. R. , J. Płotka‐Wasylka , and M. Locatelli . 2024. “Modified GAPI (MoGAPI) Tool and Software for the Assessment of Method Greenness: Case Studies and Applications.” Analytica 5, no. 3: 451–457. 10.3390/analytica5030030.

[bmc70529-bib-0044] Meister, I. , P. Zhang , A. Sinha , et al. 2021. “High‐Precision Automated Workflow for Urinary Untargeted Metabolomic Epidemiology.” Analytical Chemistry 93, no. 12: 5248–5258. 10.1021/acs.analchem.1c00203. 33739820 PMC8041248

[bmc70529-bib-0045] Miura, K. , K. Sakata , M. Someya , et al. 2012. “The Combination of Olaparib and Camptothecin for Effective Radiosensitization.” Radiation Oncology 7, no. 1: 62. 10.1186/1748-717X-7-62. 22524618 PMC3430568

[bmc70529-bib-0046] Mu, L. , Q. Liu , S. Sun , et al. 2025. “Stability Analysis of Sodium Saccharin in Fried Nuts and Seeds—Determination of Sodium Saccharin and o‐Sulfamoylbenzoic Acid by HPLC.” Frontiers in Nutrition 12: 1645604. 10.3389/fnut.2025.1645604. 41211207 PMC12593504

[bmc70529-bib-0047] Nowak, P. M. , W. Wojnowski , N. Manousi , V. Samanidou , and J. Płotka‐Wasylka . 2025. “Red Analytical Performance Index (RAPI) and Software: The Missing Tool for Assessing Methods in Terms of Analytical Performance.” Green Chemistry 27, no. 19: 5546–5553. 10.1039/D4GC05298F.

[bmc70529-bib-0048] Ottria, R. , A. Ravelli , M. Miceli , S. Casati , M. Orioli , and P. Ciuffreda . 2019. “Quantitative Characterization of Olaparib in Nanodelivery System and Target Cell Compartments by LC‐MS/MS.” Molecules 24, no. 5: 989. 10.3390/molecules24050989. 30862103 PMC6429415

[bmc70529-bib-0049] Palazzo, A. , C. Ciccarese , R. Iacovelli , et al. 2023. “Major Adverse Cardiac Events and Cardiovascular Toxicity With PARP Inhibitors‐Based Therapy for Solid Tumors: A Systematic Review and Safety Meta‐Analysis.” ESMO Open 8, no. 2: 101154. 10.1016/j.esmoop.2023.101154. 36893518 PMC10163166

[bmc70529-bib-0050] Pantziarka, P. , G. Bouche , V. Sukhatme , L. Meheus , I. Rooman , and V. P. Sukhatme . 2016. “Repurposing Drugs in Oncology (ReDO)‐Propranolol as an Anti‐Cancer Agent.” Ecancermedicalscience 10: 680. 10.3332/ecancer.2016.680. 27899953 PMC5102691

[bmc70529-bib-0051] Patakidou, C. , M. Ntorkou , and C. K. Zacharis . 2025. “Sustainable Microextraction Using Switchable Solubility Solvent for the Liquid Chromatographic Determination of Three Profenoid Drugs in Urine Samples.” Journal of Separation Science 48, no. 7: e70223. 10.1002/jssc.70223. 40650458 PMC12254904

[bmc70529-bib-0052] Polson, C. , P. Sarkar , B. Incledon , V. Raguvaran , and R. Grant . 2003. “Optimization of Protein Precipitation Based Upon Effectiveness of Protein Removal and Ionization Effect in Liquid Chromatography–Tandem Mass Spectrometry.” Journal of Chromatography B 785, no. 2: 263–275. 10.1016/S1570-0232(02)00914-5. 12554139

[bmc70529-bib-0053] Pramanik, S. , A. S. M. Islam , I. Ghosh , and P. Ghosh . 2024. “Supramolecular Chemistry of Liquid–Liquid Extraction.” Chemical Science 15, no. 21: 7824–7847. 10.1039/D4SC00933A. 38817569 PMC11134359

[bmc70529-bib-0054] Rawa‐Adkonis, M. , L. Wolska , A. Przyjazny , and J. Namieśnik . 2006. “Sources of Errors Associated With the Determination of PAH and PCB Analytes in Water Samples.” Analytical Letters 39, no. 11: 2317–2331. 10.1080/00032710600755793.

[bmc70529-bib-0055] Rekhi, G. S. , S. S. Jambhekar , P. F. Souney , and D. A. Williams . 1995. “A Fluorimetric Liquid Chromatographic Method for the Determination of Propranolol in Human Serum/Plasma.” Journal of Pharmaceutical and Biomedical Analysis 13, no. 12: 1499–1505. 10.1016/0731-7085(95)01575-2. 8788135

[bmc70529-bib-0056] Salina, E. , and L. Regazzoni . 2024. “Protein Precipitation by Metal Hydroxides as a Convenient and Alternative Sample Preparation Procedure for Bioanalysis.” Molecules 30, no. 1: 2. 10.3390/molecules30010002. 39795059 PMC11721841

[bmc70529-bib-0057] Sandré, F. , R. Moilleron , C. Morin , and L. Garrigue‐Antar . 2024. “Comprehensive Analysis of a Widely Pharmaceutical, Furosemide, and Its Degradation Products in Aquatic Systems: Occurrence, Fate, and Ecotoxicity.” Environmental Pollution 348: 123799. 10.1016/j.envpol.2024.123799. 38527585

[bmc70529-bib-0058] Šatínský, D. , V. Sobek , I. Lhotská , and P. Solich . 2019. “Micro‐Extraction by Packed Sorbent Coupled On‐Line to a Column‐Switching Chromatography System—A Case Study on the Determination of Three Beta‐Blockers in Human Urine.” Microchemical Journal 147: 60–66. 10.1016/j.microc.2019.02.069.

[bmc70529-bib-0059] Shukla, A. C. , J. M. Steven , and F. X. McGowan . 2009. “Cardiac Physiology and Pharmacology.” In A Practice of Anesthesia for Infants and Children, 361–395. Elsevier. 10.1016/B978-141603134-5.50020-2.

[bmc70529-bib-0060] Soltani, S. , and A. Jouyban . 2010. “Optimization and Validation of an Isocratic HPLC‐UV Method for the Simultaneous Determination of Five Drugs Used in Combined Cardiovascular Therapy in Human Plasma.” Asian Journal of Chemistry 23, no. 4: 1728–1734.

[bmc70529-bib-0061] Speers, A. G. , B. A. Lwaleed , J. M. Featherstone , B. J. Sallis , and A. J. Cooper . 2006. “Furosemide Reverses Multidrug Resistance Status in Bladder Cancer Cells In Vitro.” Journal of Clinical Pathology 59, no. 9: 912–915. 10.1136/jcp.2005.033100. 16556663 PMC1860466

[bmc70529-bib-0062] Tang, Y. Q. , and N. Weng . 2013. “Salting‐Out Assisted Liquid–Liquid Extraction for Bioanalysis.” Bioanalysis 5, no. 12: 1583–1598. 10.4155/bio.13.117. 23795935

[bmc70529-bib-0063] Thakur, A. , V. Saradhi Mettu , D. K. Singh , and B. Prasad . 2023. “Effect of Probenecid on Blood Levels and Renal Elimination of Furosemide and Endogenous Compounds in Rats: Discovery of Putative Organic Anion Transporter Biomarkers.” Biochemical Pharmacology 218: 115867. 10.1016/j.bcp.2023.115867. 37866801 PMC10900896

[bmc70529-bib-0064] Thummar, M. , B. S. Kuswah , G. Samanthula , U. Bulbake , J. Gour , and W. Khan . 2018. “Validated Stability Indicating Assay Method of Olaparib: LC‐ESI‐Q‐TOF‐MS/MS and NMR Studies for Characterization of Its New Hydrolytic and Oxidative Forced Degradation Products.” Journal of Pharmaceutical and Biomedical Analysis 160: 89–98. 10.1016/j.jpba.2018.07.017. 30075398

[bmc70529-bib-0065] Viode, A. , P. Van Zalm , K. K. Smolen , et al. 2023. “A Simple, Time‐and‐Cost‐Effective, High‐Throughput Depletion Strategy for Deep Plasma Proteomics.” Science Advances 9, no. 13: eadf9717. 10.1126/sciadv.adf9717. 36989362 PMC10058233

[bmc70529-bib-0066] Wenk, M. , L. Haegeli , H. Brunner , and S. Krähenbühl . 2006. “Determination of Furosemide in Plasma and Urine Using Monolithic Silica Rod Liquid Chromatography.” Journal of Pharmaceutical and Biomedical Analysis 41, no. 4: 1367–1370. 10.1016/j.jpba.2006.02.025. 16569489

[bmc70529-bib-0067] Wishart, D. S. , A. Guo , E. Oler , et al. 2022. “HMDB 5.0: The Human Metabolome Database for 2022.” Nucleic Acids Research 50, no. D1: D622–D631. 10.1093/nar/gkab1062. 34986597 PMC8728138

[bmc70529-bib-0029] World Health Organization . 2025. “Hypertension.” Accessed November 27, 2025. https://www.who.int/news‐room/fact‐sheets/detail/hypertension.

[bmc70529-bib-0068] Zhao, J. , H.‐L. Wu , J.‐F. Niu , et al. 2012. “Chemometric Resolution of Coeluting Peaks of Eleven Antihypertensives From Multiple Classes in High Performance Liquid Chromatography: A Comprehensive Research in Human Serum, Health Product and Chinese Patent Medicine Samples.” Journal of Chromatography B 902: 96–107. 10.1016/j.jchromb.2012.06.032. 22795572

[bmc70529-bib-0069] Zhu, H. , Y. Tian , H. Chen , et al. 2025. “Targeting DNA Damage Response Pathways in Tumor Drug Resistance: Mechanisms, Clinical Implications, and Future Directions.” Drug Resistance Updates 83: 101287. 10.1016/j.drup.2025.101287. 40795793

