# Supplementary material for: Optimized SPE‐HPLC‐FLD Method for the Simultaneous Determination of Olaparib, Propranolol, and Furosemide in Human Urine
Source: Biomed Chromatogr. 2026 Jun 26;40(8):e70529. doi: 10.1002/bmc.70529 (PMC13306917; doi:10.1002/bmc.70529)
Supplement: Supplementary file 1 — Figure S1: Chemical structure of OLA, PRP, and FUR. Table S1: Physicochemical properties. Figure S2: Chromatogram of FUR, PRP, and OLA using MeOH‐NaH2PO4 20 mM, pH = 3.6 45:55 v/v, as the mobile phase (isocratic elution, flow rate 0.8 mL/min) and a Nucleosil Macherey‐Nagel C8 (250 mm × 4.6 mm, 5 μm), 50°C as stationary (black) and blank chromatogram (pink). Figure S3: Chromatogram of FUR, PRP, and OLA using MeOH‐NaH2PO4 20 mM, pH = 3.6, 45:55 v/v, as the mobile phase (gradient elution, flow rate 1 mL/min) and a Nucleosil Macherey‐Nagel C8 (250 mm × 4.6 mm, 5 μm), 45°C, as stationary (black) and blank chromatogram (pink), at HPLC‐UV instrumentation. Table S2: Method FLD RF‐20A conditions. Table S3: Sample processing conditions and %recovery results. Table S4: Optimal fitting models for the selected responses (analysis of variance). Table S5: Polynomial model equations for the selected responses. Figure S4: Predicted vs. actual values of FUR, PRP, and OLA. [file BMC-40-e70529-s001.docx]

**Supplementary Information**

**Optimized SPE-HPLC-FLD Method for the Simultaneous Determination of Olaparib, Propranolol, and Furosemide in Human Urine**

Georgios Kamaris^1^ Antonia Kalagia^1^ and Catherine K. Markopoulou ^*,1^

^1^ Laboratory of Pharmaceutical Analysis, Department of Pharmacy, Aristotle University of Thessaloniki, 54124 Thessaloniki, Greece; [kamarisg@pharm.auth.gr](mailto:kamarisg@pharm.auth.gr); akalagic@pharm.auth.gr; [amarkopo@pharm.auth.gr](mailto:amarkopo@pharm.auth.gr)

^*^Corresponding Author: amarkopo@pharm.auth.gr Tel.: (+30) 2310 997665


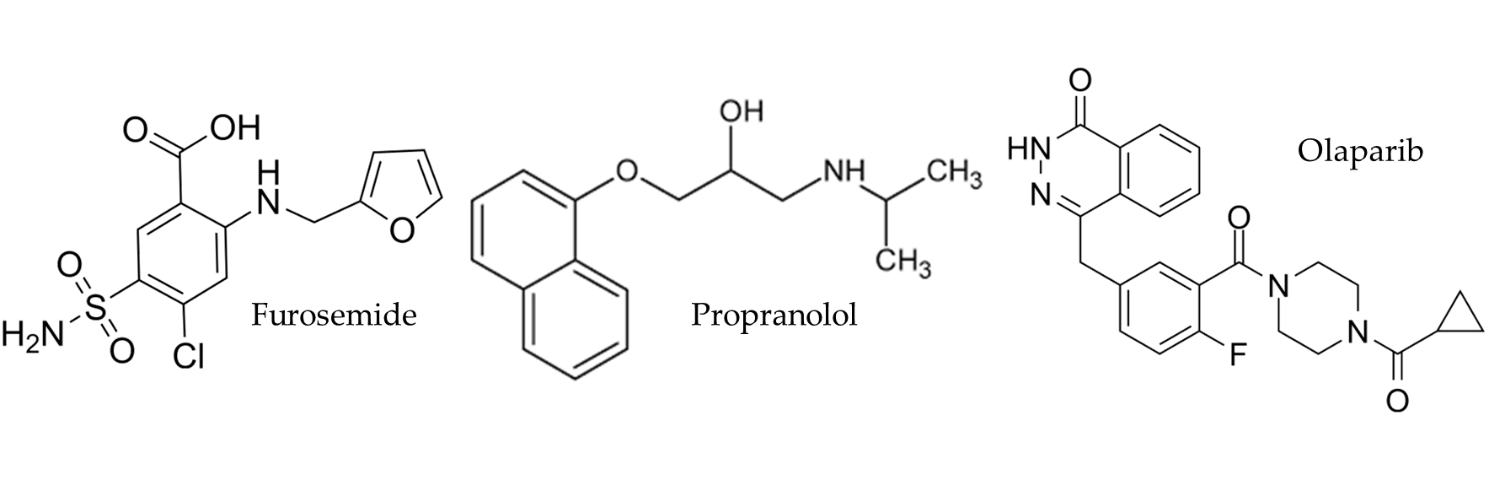


Figure S1. Chemical structure of OLA, PRP, FUR.

Table S1. Physicochemical properties.

|  | Olaparib | Propranolol | Furosemide |
| --- | --- | --- | --- |
| Molecular Weight (g/mol) | 435.5 | 259.34 | 330.74 |
| Log P | 1.9 | 3 | 2 |
| pka (strongest acidic) | 8.75 | 14.09 | 4.25 |
| pka (strongest basic) | 2.21 | 9.67 | -1.5 |
| Water Solubility (mg/ml) | Not Available | 0.062 | 0.0731 |


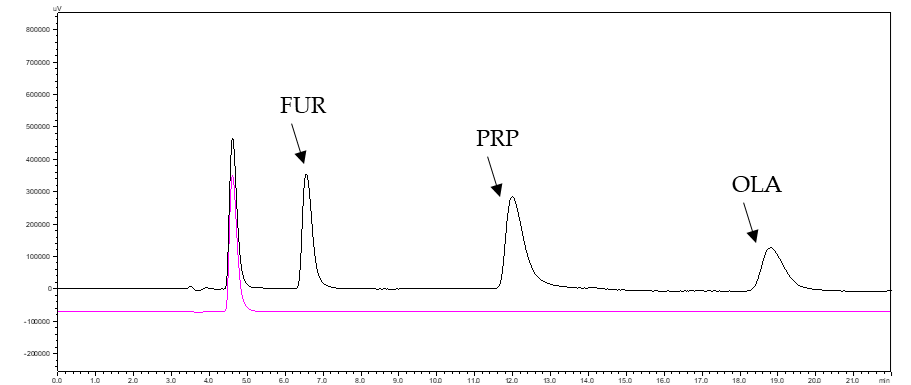


Figure S2. Chromatogram of FUR, PRP and OLA using MeOH- NaH_2_PO_4_ 20mM pH= 3.6 45:55 v/v as mobile phase (isocratict elution, flow rate 0.8mL/min) and a Nucleosil Machery-Nagel C8 (250 mm x 4.6 mm, 5μm), 50^o^C as stationery (black) and blank chromatogram (pink)


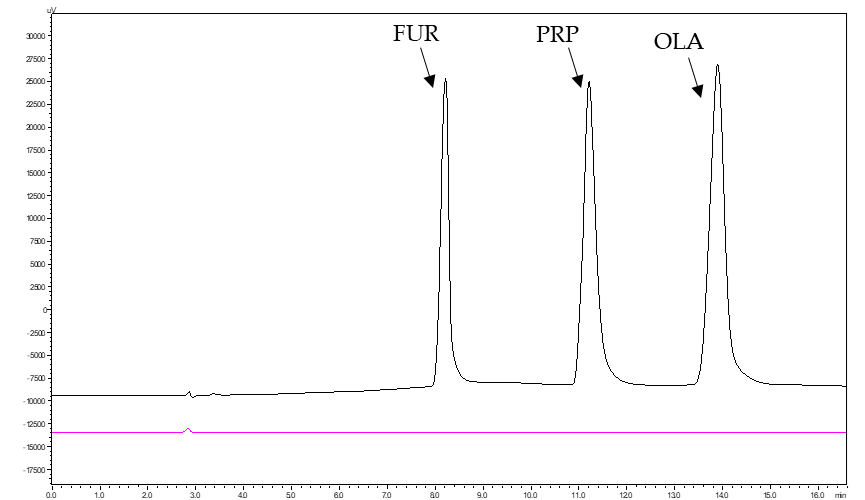


Figure S3. Chromatogram of FUR, PRP and OLA using MeOH- NaH2PO4 20mM pH= 3.6, 45:55 v/v as mobile phase (gradient elution, flow rate 1mL/min) and a Nucleosil Machery-Nagel C8 (250 mm x 4.6 mm, 5μm), 45 ^o^C as stationery (black) and blank chromatogram (pink), at HPLC-UV instrumentation.

Table S2. Method FLD RF-20A Conditions

| Channel 1 exc 327nm and em 412nm  Channel 2 exc 236 nm and em 675 nm |
| --- |
| 17.00 RF-20A(DET.A) Emission Wavelength: 350nm  17.00 RF-20A(DET.A) Emission Wavelength Ch2: 355nm  17.00 RF-20A(DET.A) Excitation Wavelength: 240 nm  17.00 RF-20A(DET.A) Excitation Wavelength Ch2: 280 nm  28.00 Controller Stop |

Table S3. Sample processing conditions and %recovery results.

|  | Component 1 | Component 2 | Component 3 | Factor 4 | Responses | | |
| --- | --- | --- | --- | --- | --- | --- | --- |
| Run | A:MeOH | B:ACN | C:ISO | D:pH elution | FUR | PRP | OLA |
|  | (mL) | (mL) | (mL) |  | % Recovery | | |
| 1 | 1 | 0 | 0 | 3 | 87 | 61 | 93 |
| 2 | 0 | 0 | 1 | 10 | 96 | 99 | 88 |
| 3 | 0.33 | 0.33 | 0.33 | 6 | 95 | 103 | 103 |
| 4 | 0 | 1 | 0 | 10 | 12 | 37 | 0 |
| 5 | 0.33 | 0.33 | 0.33 | 8 | 85 | 86 | 89 |
| 6 | 0.33 | 0.33 | 0.33 | 4 | 99 | 103 | 104 |
| 7 | 0 | 1 | 0 | 3 | 12 | 34 | 33 |
| 8 | 0.33 | 0.33 | 0.33 | 6 | 101 | 101 | 100 |
| 9 | 0 | 0 | 1 | 6 | 88 | 88 | 87 |
| 10 | 0 | 1 | 0 | 3 | 20 | 41 | 40 |
| 11 | 0 | 0 | 1 | 3 | 87 | 87 | 85 |
| 12 | 0 | 1 | 0 | 10 | 17 | 40 | 5 |
| 13 | 0.33 | 0.33 | 0.33 | 8 | 102 | 97 | 96 |
| 14 | 0.33 | 0.33 | 0.33 | 10 | 98 | 94 | 81 |
| 15 | 0.33 | 0.33 | 0.33 | 3 | 93 | 87 | 88 |
| 16 | 0.33 | 0.33 | 0.33 | 8 | 84 | 79 | 77 |
| 17 | 0 | 1 | 0 | 6 | 22 | 45 | 45 |
| 18 | 1 | 0 | 0 | 10 | 71 | 83 | 83 |
| 19 | 0.33 | 0.33 | 0.33 | 10 | 73 | 85 | 50 |
| 20 | 0.33 | 0.33 | 0.33 | 10 | 71 | 84 | 72 |
| 21 | 0 | 0 | 1 | 3 | 73 | 86 | 91 |
| 22 | 1 | 0 | 0 | 6 | 73 | 85 | 96 |
| 23 | 0.33 | 0.33 | 0.33 | 3 | 71 | 82 | 87 |
| 24 | 0.33 | 0.33 | 0.33 | 4 | 71 | 82 | 87 |
| 25 | 0.33 | 0.33 | 0.33 | 3 | 70 | 83 | 87 |
| 26 | 1 | 0 | 0 | 10 | 73 | 83 | 65 |
| 27 | 1 | 0 | 0 | 3 | 77 | 87 | 92 |
| 28 | 0.33 | 0.33 | 0.33 | 6 | 75 | 85 | 90 |

Table S4. Optimal fitting models for the selected responses (Analysis of Variance)

| Response | 1: FUR | |  |
| --- | --- | --- | --- |
| Reduced Quadratic x Mean model | | | |
| Source | F-value | | p-value |
| Model | 48.65 | | < 0.0001 |
| ⁽¹⁾Linear Mixture | 59.42 | | < 0.0001 |
| AB | 36.29 | | < 0.0001 |
| BC | 41.31 | | < 0.0001 |
| Response | 2: PRP | |  |
| Reduced Quadratic x Mean model | | | |
| Source | F-value | p-value | |
| Model | 42.78 | < 0.0001 | |
| ⁽¹⁾Linear Mixture | 54.48 | < 0.0001 | |
| AB | 30.57 | < 0.0001 | |
| BC | 33.10 | < 0.0001 | |
| Response | 3: OLA | |  |
| Reduced Quadratic x Quadratic model | | | |
| Source | F-value | | p-value |
| Model | 76.80 | | < 0.0001 |
| ⁽¹⁾Linear Mixture | 176.30 | | < 0.0001 |
| AB | 26.82 | | < 0.0001 |
| AD | 18.94 | | 0.0003 |
| BC | 77.11 | | < 0.0001 |
| BD | 44.12 | | < 0.0001 |
| AD² | 13.29 | | 0.0017 |
| BD² | 28.97 | | < 0.0001 |

Table S5: Polynomial model equations for the selected responses.

FUR = +77.14442 MeOH + 17.30610 ACN + 85.55419 ISO + 14865469 MeOH * ACN + 159.40667 ACN * ISO

PRP = +81.87871 MeOH + 39.82018 ACN +91.48966 ISO +106.21399 MeOH * ACN +111.09552 ACN * ISO

OLA = +56.77906 MeOH -16.63127 ACN +87.93029 ISO +75.69759 MeOH * ACN +16.89717 MeOH * pH elution +128.10181 ACN * ISO +24.44118 ACN * pH elution 1.54277 MeOH * pH elution² -2.24665 ACN * pH elution²


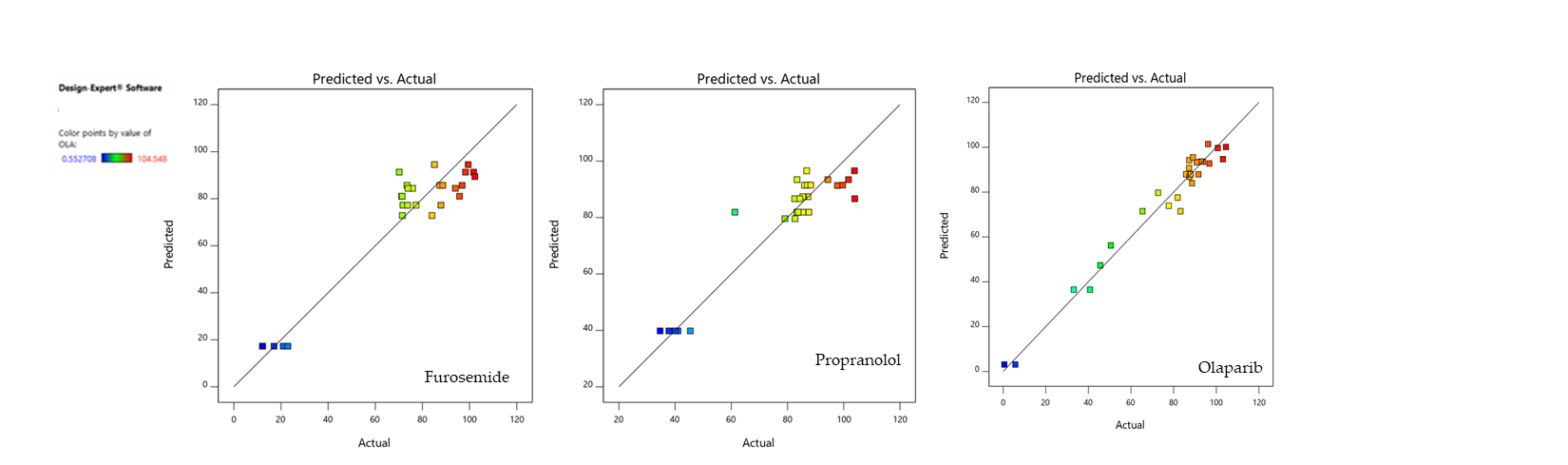


Figure S4. Predicted vs actual values of FUR, PRP, OLA.
